# Space-efficient computation of *k*-mer dictionaries for large values of *k*

**DOI:** 10.1186/s13015-024-00259-1

**Published:** 2024-04-05

**Authors:** Diego Díaz-Domínguez, Miika Leinonen, Leena Salmela

**Affiliations:** Department of Computer Science, University of Helsinki, Pietari Kalmin katu 5, 00014 Helsinki, Finland

**Keywords:** Genomics, String hashing, *k*-mers

## Abstract

Computing *k*-mer frequencies in a collection of reads is a common procedure in many genomic applications. Several state-of-the-art *k*-mer counters rely on hash tables to carry out this task but they are often optimised for small *k* as a hash table keeping keys explicitly (i.e., *k*-mer sequences) takes $O(N\frac{k}{w})$ computer words, *N* being the number of distinct *k*-mers and *w* the computer word size, which is impractical for long values of *k*. This space usage is an important limitation as analysis of long and accurate HiFi sequencing reads can require larger values of *k*. We propose Kaarme, a space-efficient hash table for *k*-mers using $O(N+u\frac{k}{w})$ words of space, where *u* is the number of reads. Our framework exploits the fact that consecutive *k*-mers overlap by $k-1$ symbols. Thus, we only store the last symbol of a *k*-mer and a pointer within the hash table to a previous one, which we can use to recover the remaining $k-1$ symbols. We adapt Kaarme to compute canonical *k*-mers as well. This variant also uses pointers within the hash table to save space but requires more work to decode the *k*-mers. Specifically, it takes $O(\sigma ^{k})$ time in the worst case, $\sigma$ being the DNA alphabet, but our experiments show this is hardly ever the case. The canonical variant does not improve our theoretical results but greatly reduces space usage in practice while keeping a competitive performance to get the *k*-mers and their frequencies. We compare canonical Kaarme to a regular hash table storing canonical *k*-mers explicitly as keys and show that our method uses up to five times less space while being less than 1.5 times slower. We also show that canonical Kaarme uses significantly less memory than state-of-the-art *k*-mer counters when they do not resort to disk to keep intermediate results.

## Introduction

Strings of length *k* called *k*-mers are central in many genomic analyses. While in the past, relatively short *k*-mers with *k* less than 100 have been used to analyse short read data produced by Illumina sequencers, the development of long and very low error rate sequencing technologies such as HiFi sequencing by Pacific Biosciences has created a need to support longer *k*-mers to take advantage of the length of the reads. For example, when assembling this data to whole genomes using de Bruijn graphs, assemblers use *k*-mers with *k* up to several thousand base pairs to decrease branching in the de Bruijn graph [2, 25]. A hash table associating *k*-mers to values such as the frequency of a *k*-mer is a basic data structure used in the analysis of *k*-mers. This work presents a space-efficient hash table for long *k*-mers.

The use of *k*-mers is common in many tasks in the analysis of sequencing data including error correction [7, 13], genome assembly [11, 21], genetic variant calling [20, 30], metagenomic classification [32], and repeat analysis [6, 15]. The first step in most *k*-mer-based methods is counting [17], where the aim is to compute the frequencies of all *k*-mers occurring in a sequencing read set. One approach to *k*-mer counting, taken e.g. by Jellyfish [18], DSK [26], and CHTKC [31], is to use a hash table to associate the *k*-mers with their frequencies. In addition to counting, *k*-mer hash tables are useful in applications where *k*-mers have to be associated with values and random access to the *k*-mers is needed.

The hash tables used by tools like Jellyfish [18] and CHTKC [31] are highly optimised for short *k*-mers. A *k*-mer of DNA can be encoded in 2*k* bits and thus the *k*-mer can be stored in each hash table entry when $k\le 32$, i.e. the sequence fits the computer word of modern computer architectures. However, when *k* becomes large, this approach is space-consuming as the space complexity is $O(N\frac{k}{w})$ words, where *N* is the number of unique *k*-mers and *w* is the computer word size. For example, in de-Bruijn-graph-based genome assemblers for HiFi reads, it is crucial to use a large value of *k* to take advantage of the read lengths and decrease branching in the de Bruijn graph. However, due to the memory bottleneck caused by long *k*-mers, these tools resort to various ways to avoid creating a dictionary of all *k*-mers in the read set. For instance, MBG (Minimizer-based sparse de Bruijn Graph) [25] uses minimizer winnowing [27] to choose a subset of the *k*-mers and then builds a sparse de Bruijn graph using this subset.

LJA (La Jolla Assembler) [2] uses a complex procedure to directly construct the compressed de Bruijn graph, which is a memory-efficient version of a de Bruijn graph where all nonbranching paths have been compressed. LJA first builds a sparse de Bruijn graph using minimizers similar to MBG. The sparse de Bruijn graph is then used to create a set of disjointigs, a set of strings containing all *k*-mers of the original read set as substrings. Then a Bloom-filter is used to record all *k*-mers present in the disjointigs and finally the compressed de Bruijn graph is built.

We propose Kaarme,^1^ a space-efficient hash table for *k*-mers that uses $O(N + u\frac{k}{w})$ words of space, where *u* is the number of strings in the input data set. Kaarme is an in-memory data structure that stores for each hash table entry the last symbol of the *k*-mer, a pointer to a previous *k*-mer, and some bookkeeping bits. A key insight in Kaarme is that a common pattern in many applications is to access the entries so that each *k*-mer overlaps by $k-1$ symbols with the previous one. This allows for fast detection of collisions in our scheme in most cases as it suffices to check that the pointer in the hash table entry matches the pointer of the previous *k*-mer and that the last symbol of the *k*-mer matches the symbol saved in the hash table entry. Additionally, we show how the hash table can be adapted to store only canonical *k*-mers, i.e. *k*-mers that are smaller than their reverse complements in some predefined order, and we give a lock-free parallel implementation of the hash table and its construction.

We compare canonical Kaarme against a regular hash table storing the whole *k*-mer’s canonical sequence in each entry and show that Kaarme uses up to five times less space than a regular hash table while being at most 1.5 times slower. Additionally, we compare canonical Kaarme against *k*-mer counters that rely on hash tables and show that, for data sets where the *k*-mer counters do not resort to disk space, Kaarme uses significantly less RAM.

## Related work

The problem of constructing a compact representation for an input set of *k*-mers has been studied before. These solutions typically also take advantage of the *k*-mers sharing long overlaps with each other. BOSS [4] uses a data structure that resembles the Burrows–Wheeler transform to represent a de Bruijn graph, i.e. a set of *k*-mers. UST [24] and ProphASM [5] find a small spectrum preserving string set (SPSS) which is a set of strings containing all *k*-mers in the input *k*-mer set and Eulertigs [28] gives a minimum SPSS. Shibuya et al. [29] consider compressing a *k*-mer count table using compressed static functions and minimizers. Finally, Pibiri [22] and Pibiri et al. [23] consider the problem of associating each *k*-mer in a *k*-mer set with a unique integer in the range $[1\ldots n]$ where *n* is the number of *k*-mers. However, all these tools require that the set of *k*-mers has already been counted, which is exactly what Kaarme hash table is designed for.

Bifrost [10] can directly compute the compacted de Bruijn graph from input reads. However, it outputs the compacted de Bruijn graph, which does not allow values to be associated with single *k*-mers like our hash table. Methods such as TwoPaCo [19] and Cuttlefish [14] compute a de Bruijn graph for genomic sequences. These tools exploit the length of the sequences and the fact that all *k*-mers will be kept in the final data structure, unlike when working with reads, where *k*-mers with low counts are often discarded.

Some *k*-mer counters [8, 9, 16] exploit the overlaps between *k*-mers by using super *k*-mers and minimizers. A minimizer of a string is the smallest substring of a given length in some predefined order, and a super *k*-mer is a string consisting of consecutive *k*-mers that share the same minimizer. These *k*-mer counters first split the reads into super *k*-mers, and the super *k*-mers are then partitioned into bins so that the super *k*-mers sharing the same minimizer end up in the same bin. Since all *k*-mers with the same minimizer are now in the same bin, different bins do not share any *k*-mers and can thus be counted independently, allowing for efficient parallelization.

## Preliminaries

### De Bruijn graphs

The order *k* de Bruijn graph (dBG) $G=(V, E)$ of a string $S[1\ldots n]$ over the alphabet $\Sigma$ is a labelled directed graph that encodes the distinct *k*-length substrings of *S*. Each node $v \in V$ is labelled with one of the distinct *k*-length substrings. Thus, if *S* has *N* distinct *k*-length substrings, *G* has *N* nodes. Additionally, two nodes *u* and *v* are connected by an edge $(u, v) \in E$ if the $k-1$-length suffix of *label*(*u*) matches the $k-1$-length prefix of *label*(*v*), and the collapse of the overlap yields a $k+1$-length sequence that exists as a substring in *S*. The label of (*u*, *v*) is the rightmost symbol of *label*(*v*).

### String fingerprints and rolling hashing

The Karp–Rabin method [12] computes fingerprints (integer values) for strings of arbitrary size. Given a sequence $K[1\ldots k]$ over the alphabet $\Sigma$, a fingerprint function $h_{p}: \Sigma ^{k} \rightarrow [1\ldots p]$ is defined as

$$\begin{aligned} h_{p}(K[1\ldots k]) = \left( \sum _{i=1}^{k} K[i]\cdot q^{i-2}\right) \bmod p, \end{aligned}$$

where $q \in [1\ldots p]$ is an integer chosen uniformly at random and *p* is a prime number. Karp–Rabin fingerprints can be updated in constant time. Given $h_{p}(K[1\ldots k])$ and a symbol $c \in \Sigma$, one can compute $h_{p}(K[2\ldots k]{\cdot }{c})$ or $h_{p}(c{\cdot }K[1\ldots k-1])$ in *O*(1) time (among other operations). The fast update makes Karp–Rabin fingerprints the preferred solution to implement rolling hashing in linear time. That is, given a string $T[1\ldots n]$ and an integer $k<n$, compute the fingerprint $h_{p}(T[i\ldots i+k-1])$ of every *k*-length substring $T[i\ldots i+k-1]$, with $i \in [1\ldots n-k+1]$.

To insert the strings visited by the rolling hash into a hash table of size *m*, it is convenient to combine $h_{p}$ with a universal hash function $h_{m}: [1\ldots p] \rightarrow [1\ldots m]$ using the composition $h(K)=h_m(h_p(K))$ with $m<p$. By using a large prime *p*, the probability for two random strings over $\Sigma ^{k}$ to be assigned the same integer in $[1\ldots m]$ is close to 1/*m*.

## Our contribution

### Definitions

We consider the RAM model of computation. Given an input of *n* symbols, we assume our procedures run in random-access memory, where the machine words are $w=\Theta (\log n)$ bits in length and can be manipulated in constant time.

Let $\{\texttt {a}, \texttt {c}, \texttt {g}, \texttt {t}\}$ be the DNA *alphabet*, and let $\Sigma =\{0, 1, 2, 3, 4\}$ be another set of size $\sigma =|\Sigma |=5$ to which we map the DNA alphabet as $\texttt {a}=1$, $\texttt {c}=2$, $\texttt {g}=3$, $\texttt {t}=4$. For technical reasons, we define $\varepsilon =0\in \Sigma$ as the empty symbol. Additionally, we regard the DNA *complement* as a permutation $\pi [1,\sigma ]$ that reorders the symbols in $\Sigma$, exchanging $1=\texttt {a}$ with $4=\texttt {t}$, $2=\texttt {c}$ with $3=\texttt {g}$, and keeping the same value $\pi [\varepsilon ] = \varepsilon$ for the empty symbol. The *reverse complement* of $R \in \Sigma ^{*}$, denoted $\hat{R}$, is the string formed by reversing *R* and replacing every symbol *R*[*j*] by its complement $\pi (R[j])$.

Given two strings $K, K' \in \Sigma ^{k}$, the operator $K \oplus K'$ means that the $k-1$-length suffix $K[2\ldots k]$ overlaps the $k-1$-length prefix $K'[1\ldots k-1]$.

We consider a collection $\mathcal {R}=\{R_1, \ldots , R_{u} \}$ of *u* strings over the alphabet $\Sigma ^{*}$, with a total length of $n = ||\mathcal {R}|| =\sum ^{u}_{i=1} |R_{i}|$ symbols.

Let $h_{p}: \Sigma ^{k} \rightarrow [0\ldots p-1]$ be a function that maps *k*-length strings to integers in $[0\ldots p-1]$ uniformly at random, where *p* is a prime number. We define the *canonical* version of a *k*-mer *K*, denoted $K^{c}$, to be the string $K^{c} \in \{K, \hat{K}\}$ with the smallest value in $h_{p}$. If $h_{p}$ assigns the same integer to *K* and $\hat{K}$, we set $K^{c}$ equal to the smallest string in lexicographical order between *K* and $\hat{K}$.

A *k*-mer dictionary $\mathcal {D}_{k,\mathcal {R}}$ is a dictionary where the keys are the distinct *k*-length substrings of $\mathcal {R}$ (i.e., the *k*-mers), and their associated values are the frequencies of those *k*-mers in $\mathcal {R}$. The canonical *k*-mer dictionary $\mathcal {D}_{k,\mathcal {R}}^{c}$ stores as keys the canonical *k*-mers of $\mathcal {R}$. The value associated with each key $K^{c}$ in $\mathcal {D}^{c}_{k,\mathcal {R}}$ is the sum of the frequencies in $\mathcal {R}$ for *K* and $\hat{K}$.

Our framework consists of three algorithms:

1. GetDict$(\mathcal {R}, k)$ (“Building the dictionary” section): recieves $\mathcal {R}$ as input and returns a hash table *H* encoding $\mathcal {D}_{k,\mathcal {R}}$ in compressed form.
2. GetCanDict$(\mathcal {R}, k)$ (“Building the canonical dictionary” section): receives $\mathcal {R}$ as input and returns a hash table *H* encoding $\mathcal {D}^{c}_{k,\mathcal {R}}$ in compressed form.
3. DumpDict(*H*) (“Reporting the *k*-mers in the compact dictionary” section): receives as input a hash table *H* encoding a *k*-mer dictionary (canonical or non-canonical) in compressed form and returns the same dictionary in uncompressed form. That is, each entry (*K*, *f*) of key *K* and frequency *f* is represented as a string in $\Sigma ^{k}$ and an integer, respectively.

Our theoretical descriptions in the following sections assume a suitable size *m* for *H* is known prior to the execution of GetDict or GetCanDict. A suitable *m* is big enough to encode the distinct *k*-mers of $\mathcal {R}$. Let *H* be a hash table that uses open addressing to resolve collisions and *h* a hash function that maps *k*-mers to buckets in *H*. We define the following operations:

1. *incval*(*H*, *K*, *f*): increments the value associated with the key *K* in *H* by *f* or stores a new entry (*K*, *f*) in *H* if *K* does not exist as key.
2. *value*(*H*, *K*): returns the value associated with the key *K* in *H*.
3. *probe*(*h*(*K*), *m*, *d*): receives as input the fingerprint *h*(*K*) of a *k*-mer *K* (“String fingerprints and rolling hashing” section) and returns the hash table bucket in $[1\ldots m]$ that the probing function of the open addressing scheme produces in step *d*.

We assume *probe*(*h*(*K*), *m*, *d*) computes the bucket using quadratic probing.

### Dictionary of *k*-mers

We begin by describing how to build the *k*-mer dictionary $\mathcal {D}_{k, \mathcal {R}}$ efficiently. “Our compact hash table” section presents our compact data structure that exploits the redundancy of consecutive *k*-mer in $\mathcal {R}$. Then, in “Building the dictionary” section, we explain our algorithm GetDict, which builds $\mathcal {D}_{k, \mathcal {R}}$ using this compact data structure. “Connection with de Bruijn graphs” section describes the link between our method and the de Bruijn graph of $\mathcal {R}$. Finally, in “Space and time complexity” section, we present the space and time complexities of GetDict, and in “Improving the time complexity” section, we show how to improve its running time.

#### Our compact hash table

We devise a compact hash table *H* where the keys are the distinct *k*-mers of $\mathcal {R}$, and the associated values are their frequencies. We reduce space usage by exploiting the fact that *k*-mers occurring consecutively in $\mathcal {R}$ contain redundant information. Specifically, let $K_{pr} = R_{i}[x-1\ldots y-1]$ and $K=R_{i}[x\ldots y]$ be two consecutive substrings in $R_{i} \in \mathcal {R}$, with $k=y-x+1$ and $K_{pr} \ne K$. Our simple observation is that, storing $K_{pr}$ and *K* explicitly in *H* produces a redundant copy of the $(k-1)$-length string $R_{i}[x\ldots y-1] = K_{pr}[2\ldots k] = K[1\ldots k-1]$.

In our encoding, a bucket *H*[*b*] stores a *k*-mer *K* in relative form along with its frequency in $\mathcal {R}$. This representation has three fields $H[b]=(f, r, a)$, where *f* is the frequency of *K* in $\mathcal {R}$, *r* is another bucket in *H* where we can recover the prefix $K[1\ldots k-1]$ and $a = K[k] \in \Sigma$ is the rightmost symbol in *K*. We refer to $H[b].r = b_{pr}$ as the *reference* bucket for *K*. We also keep a dynamic buffer *B* to store the *k*-mers that we cannot encode immediately in relative form. This situation occurs when, during the execution of GetDict, we visit the *k*th prefix $K=R_{i}[1\ldots k]$ of a string $R_{i} \in \mathcal {R}$. The problem arises because we do not know a bucket $H[b_{pr}]$ encoding a *k*-mer $K_{pr}$ with $R_{i}[1\ldots k-1]=K_{pr}[2\ldots k]$ that we can record as a reference in *H*[*b*].*r*. Thus, we get the leftmost available block $B[l\ldots l+k-1]$, copy *K* there, and set $H[b] = (1,l, \varepsilon )$, where $\varepsilon$ serves as a flag that indicates that *K* is in the dynamic buffer. We say that *B* is dynamic because, as soon as we find a bucket $H[b_{pr}]$, we remove *K* from *B* and store $H[b].r=b_{pr}$ instead.

#### Building the dictionary

We now describe GetDict, our method to compute the dictionary $\mathcal {D}_{k, \mathcal {R}}$ that relies on the compact hash table *H* of “Our compact hash table” section. Algorithm 4.2.2 contains more details about its implementation.

We start the algorithm by defining a rolling hash function $h: \Sigma ^{k} \rightarrow [1\ldots m]$ that maps *k*-mers to buckets in *H* uniformly at random as described in “Preliminaries” section, and by creating an empty dynamic buffer *B*. Subsequently, we scan $\mathcal {R}$ from left to right, and for every *k*-mer $K=R[x\ldots y]$ we visit, with $R \in \mathcal {R}$, we call the operation *incval*(*H*, *K*, 1) (“Definitions” section). Lines 28–33 depict this idea.

Before describing how *incval* works, we will introduce some useful notation. Let $K = R[x\ldots y]$, with $y-x+1=k$, be the active window in the scan of $\mathcal {R}$ during the execution of GetDict. Additionally, let $b_{pr}$ be the bucket in *H* where we inserted the predecessor *k*-mer $K_{pr}=R[x-1\ldots y-1]$ (i.e., the reference bucket of *K*). We assume $b_{pr}$ is *null* if *K* is the *kth* prefix of *R*. For convenience, we also change the signature of *incval* to $incval(H, K, 1, b_{pr})=b$, where *b* is the bucket where *K* resides.

When we call $incval(H, K, 1, b_{pr})$, we probe buckets in *H* using the function *probe* (“Definitions” section) until we find one that either is empty or has a key that matches *K*. Every time we probe a non-empty bucket $H[b']$, we check if *K* matches its key in *O*(*k*) time by following bucket references recursively. We refer to this procedure as *keycomp*:

- $keycomp(H, b', K, b_{pr})$: receives as input the hash table *H*, a bucket $b'$, a *k*-mer $K \in \Sigma ^{*}$, and a (possible *null*) bucket $b_{pr}$ whose triplet $H[b_{pr}]$ encodes a *k*-mer $K_{pr} = c{\cdot }K[1\ldots k-1]$, with $c \in \Sigma$, and returns *true* if the key in $H[b']$ matches *K* or *false* otherwise

When the probing mechanism reaches an empty bucket $H[b']$, we insert *K* there, but we need to check if we have a valid reference bucket first. Thus, if $b_{pr}$ is *null*, we store $B[l\ldots l+k-1]=K$ and set $H[b']=(1, l, \varepsilon )$. On the other hand, when $b_{pr}$ is not *null*, there is an available reference bucket to recover $K[1\ldots k-1]$, so we just store the triplet $H[b'] = (1, b_{pr}, K[k])$. On the other hand, if the probing mechanism reaches a bucket $H[b']$ such that $keycomp(H, b', K, b_{pr})$ is *true*, it means *K* already exist in *H*, so we just increment $H[b'].f$ by one. Additionally, if $H[b'].a=\varepsilon$ and $b_{pr}$ is not *null*, we remove $B[l\ldots l+k-1]$ and store $H[b'].r=b_{pr}$ and $H[b'].a=K[k]$ because now we have a reference bucket $H[b_{pr}]$ where to extract $K[1\ldots k-1]$. We can flag the area $B[l\ldots l+k-1]$ as reusable and fill it again later in the scan of $\mathcal {R}$ with another *k*-mer. The pseudocode of *incval* is in Lines 10–26.

The last aspect we will discuss in this section is the implementation of $keycomp(H, b', K, b_{pr})$ (Lines 1–9). We start the execution by checking if $H[b'].r = b_{pr}$ and $K[k]=H[b'].a$. When these conditions hold, we return *true* immediately because the key of $H[b_{pr}]$ is suffixed by $K[1\ldots k-1]$. Notice we can finish the call without further symbol comparisons because *incval* is a subroutine of $\textsc {GetDict}$, and this function scans $\mathcal {R}$ from left to right. Therefore, it always hold that the bucket $H[b_{pr}]$ encodes a *k*-mer $K_{pr}$ that immediately precedes *K* in $\mathcal {R}$. On the other hand, if $b_{pr} \ne H[b'].r$, we start the decompression of $H[b']$’s key. We first compare $H[b'].a$ against *K*[*k*] and set the next bucket $b'=H[b'].r$ if they match. As a general rule, in every *ith* step, we compare $H[b'].a$ against $K[k-i+1]$ and return *false* if they differ or go to the next bucket $b'=H[b'].r$ and perform another symbol comparison. When every symbol $K[k-i+1]$ matched its corresponding symbol $H[b'].a$, with $i \in [1\ldots k]$, we can be sure that $H[b']$ encodes *K*, so $keycomp(H, b', K, b_{pr})$ returns *true*. The only exception to the procedure of *keycomp* is when we reach a bucket $H[b']$ whose key is in *B*. We can easily detect this situation because $H[b'].a=\epsilon$ is a special symbol. When this happens, we get the buffer offset $l=H[b'].r$ and compare the $k-i+1$ suffix of $B[l\ldots l+k-1]$ against the prefix $K[1\ldots k-i+1]$.

**Fig. 1:**
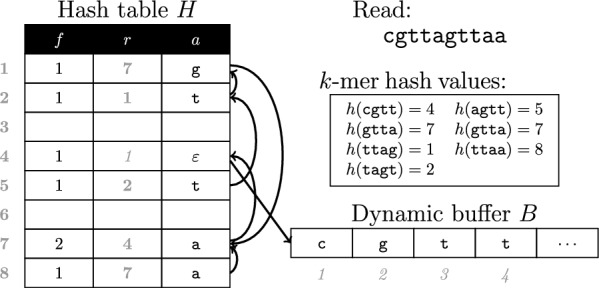
Example of our compact hash table *H* filled by GetDict using the 4-length *k*-mers of the read $R=\texttt {cgttagttaa}$. The arrows indicate the references where we recover the $(k-1)$-prefixes of the *k*-mers. We first map $R[1\ldots k] = \texttt {cgtt}$ to its bucket $H[h(\texttt {cgtt})=4]$. However, as we do not know a reference bucket $H[b_{pr}]$ to recover the prefix $R[1\ldots k-1]=\texttt {cgt}$, we store the *k*-mer’s full sequence in the dynamic buffer as $B[l=1\ldots l+k-1=4]=\texttt {cgtt}$ and store $H[4]=(f=1,r=l,a=\varepsilon )$. The next *k*-mer in $\mathcal {R}$ is $R[2\ldots k+1] = \texttt {gtta}$, whose designated bucket is $H[h(\texttt {gtta})=7]$. In this case, we have available a preceding *k*-mer $K_{pr}=\texttt {cgtt}$ and its bucket $b_{pr}=4$. Thus, we encode gtta as $H[4]=(1,4,\texttt {a})$. We continue with the remaining *k*-mers of *R* in the same way

**Algorithm 1:**
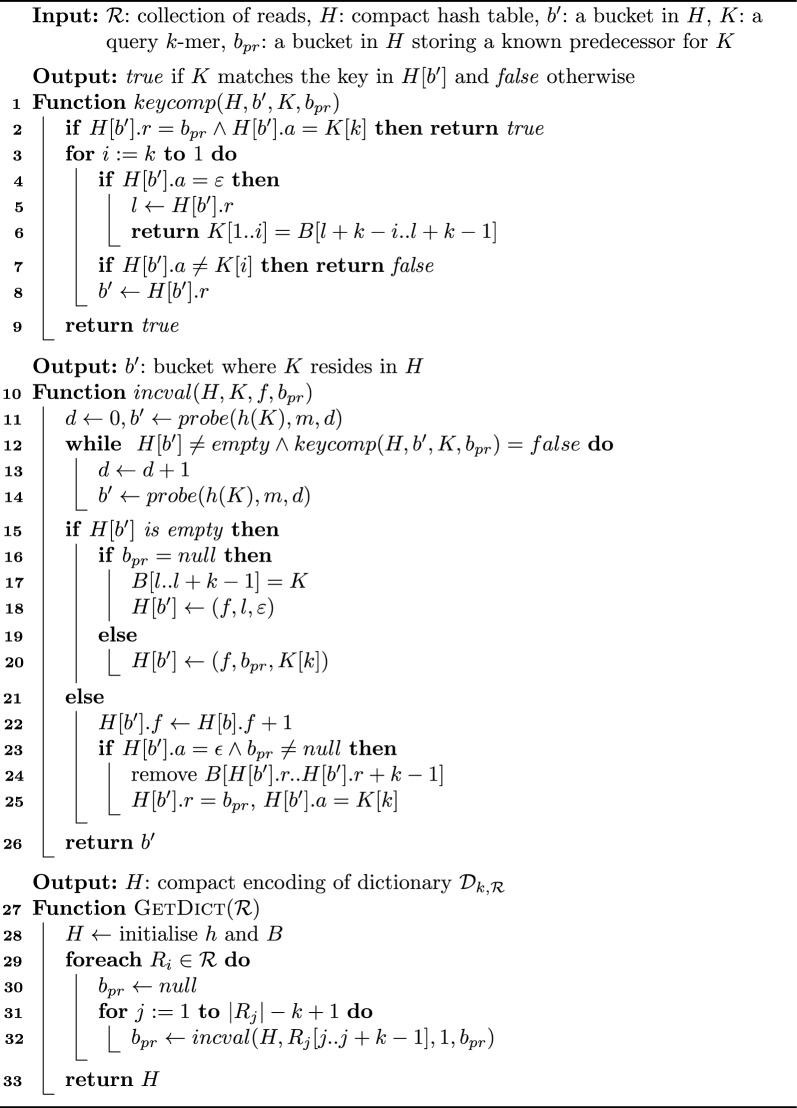
Framework of GetDict

Figure 1 shows an example of the compact hash table *H* we obtain with $\textsc {GetDict}$.

#### Connection with de Bruijn graphs

It is not difficult to see that our compact hash table resembles the de Bruijn graph. Let $G=(V, E)$ be the de Bruijn graph of order *k* obtained from $\mathcal {R}$. The pair (*H*, *B*) encodes a graph $G'=(V',E')$ that represents a sparse version of *G*, with $V'=V$ and $E' \subseteq E$. Each bucket *H*[*b*] stores a node $v \in V'$, and the field *H*[*b*].*r* represents an edge connecting *v* with one of its incoming nodes $u \in V'$. To put it differently, let *H*[*b*] be the bucket for $v \in V'$ and let $H[b']$ be the bucket for $u\ne v \in V'$. The link $H[b].r = b'$ implies the edge $(u, v) \in E'$ labelled $H[b].a \in \Sigma$. On the other hand, every node *v* encoded in *B* has indegree zero.

$G'$ is a sparse version of *G* because some edges of *E* might not be present in $E'$ since every bucket *H*[*b*] stores at most one incoming edge for *v*, and the remaining ones are ignored to save space. We remark that (*H*, *B*) offers limited navigational functionality for $G'$: each node *v* can only visit its incoming node *u* (via *H*[*b*].*r*) and retrieve the symbol *H*[*b*].*a* that labels $(u,v) \in E'$. Still, this feature is enough for us to implement *keycomp* during the probing phase of *incval*.

#### Space and time complexity

We now describe the upper bounds of our framework. We will refine these results in the following sections.

##### Theorem 1

Let $G=(V, E)$ be the de Bruijn graph of order *k* built from a collection $\mathcal {R}=\{R_{1}, \ldots , R_{u}\}$ of *u* strings and $||\mathcal {R}|| = n$ symbols, *V* being the set of nodes and *E* the set of edges. $\textsc {GetDict}(\mathcal {R}, k)$ requires $O(|V| + u\frac{k}{w})$ words of space to encode (*H*, *B*) and runs in *O*(*nk*) expected time.

##### Proof

The rolling hash function *h* allows us to get *h*(*K*) from the preceding value $h(K_{pr})$ in *O*(1) time. Thus, obtaining the buckets for all the *k*-mers in $\mathcal {R}$ takes *O*(*n*) time. When we visit *K* in $\mathcal {R}$, the probing mechanism of $incval(H, K, 1, b_{pr})$ will start to probe buckets from *H*[*h*(*K*)] until it finds one that either is empty or encodes a key matching *K*. The classical result on hash tables tells us that, by choosing a hash function *h* with collision probability 1/*m*, and setting the load factor of *H* to a constant value $\alpha$, the number of probes to find a bucket for *K* is *O*(1) in expectation. Still, every time the probing mechanism visits an occupied bucket, it has to call the function *keycomp* to compare keys, which takes *O*(*k*) time. Thus, the call of $incval(H, K,1,b_{pr})$ takes *O*(*k*) in expectation. Summing up, the complete running time of GetDict is *O*(*nk*) in expectation. *H* uses *O*(|*V*|) words of space as there is one bucket for each de Bruijn graph node $v \in V$, and there are $O(m(1-\alpha ))$ empty buckets, *m* being the hash table size. The final aspect to consider is the space usage of *B*: there are at most *u* *k*-mers in $\mathcal {R}$ that (potentially) do not have a reference in *H*, that is, the *k*th prefix of every $R_{i} \in \mathcal {R}$, with $i \in [1\ldots u]$. Assuming $\Sigma$ uses $\lceil \log \sigma \rceil = 3$ bits per symbol, then each of these *k*-mers use $\lceil 3k/w \rceil$ words, and thus the total space for *B* is $O(u\frac{k}{w})$ words. As a conclusion, the total space usage of (*H*, *B*) is $O(|V| + u\frac{k}{w})$ words. $\square$

#### Improving the time complexity

The running time *O*(*nk*) of Theorem 1 is a rather pessimistic upper bound for GetDict as $invcal(H, K, 1, b_{pr})$ does not always require *k* operations. The only case when $incval(H, K, 1, b_{pr})$ will incur in *k* symbol comparisons is when *K* is encoded in a bucket $H[b']$ with $H[b'].r\ne b_{pr}$. In that case, we need to match *K* against the key in $H[b']$ to be sure they are equal. We can express this situation in terms of de Bruijn graphs: the bucket $H[b']$ encoding the node *v* labelled $label(v)=K$ stores an incoming edge (*u*, *v*) in $H[b'].r$ that is different from the incoming edge $(u', v)$ associated with $b_{pr}$.

In an ideal scenario, where we know the set $I_{v}=\{b_1,\ldots , b_{\sigma '}\}$ of buckets in *H* storing the $\sigma ' \le \sigma$ incoming nodes of *v*, checking if an arbitrary bucket $H[b']$ encodes *v* takes *O*(1) time as the comparison of *K* against the key of $H[b']$ reduces to check if $H[b'].r \in I_{v}$. In reality, however, in the best case, we know one incoming node for *v*, i.e., the node $u'$ encoded in the bucket $H[b_{pr}]$. Still, we can get closer to the ideal scenario by increasing the space of our data structure.

We keep an auxiliary hash table $H^{e}$ that stores indexes of *H* as keys. Each key in $H^{e}$ is associated with a list of up to $\sigma -2=3$ integers. Thus, for a *k*-mer *K* encoded in the bucket *H*[*b*], the list $L = value(H^{e}, b)$ contains the buckets in $I_{v}$ that are different from *H*[*b*].*r*. We will also add a new field to the buckets of *H*. The new field $H[b].d \ge 0$ will store the number of probing steps *incval* incurred to reach *b* from *h*(*K*) when inserted *K* the first time. For example, if $probe(h(K), m, 3)=b$, then $H[b].d=3$. We also change the signature of *keycomp* to $keycomp(H, b', d, K, b_{pr})$, with $probe(h, K, d) = b'$. In other words, *d* is the number of probing steps *incval* performed to reach the bucket $H[b']$ from *H*[*h*(*K*)]. We remark that we call *keycomp* during the execution of *incval*, so we always know *d* when we call *keycomp*.

We implement $keycomp(H, b', d, K, b_{pr})$ as follows: we start by checking that $H[b'].a=K[k]$, and return *false* if they differ. Now suppose $H[b'].a=K[k]$. If $H[b'].r \ne b_{pr}$, we access the list $L = value(H^{e}, b')$ and check if one of the buckets in *L* matches $b_{pr}$. If that is the case, we return *true*. On the other hand, if $b_{pr} \notin \{L \cup H[b'].r\}$; and $|L|+2=\sigma$ or $H[b'].d \ne d$, we return *false*. When $H[b'].d=d$ and $|L|+2<\sigma$, we compare *K* against the key in $H[b']$ as usual. When they match, we store $b'$ in *L* and return *true*, otherwise we return *false*.

##### Theorem 2

Let $G=(V, E)$ be the de Bruijn graph with order *k* built from the collection $\mathcal {R}$ of *u* strings over the constant alphabet of size 4, and with a total of $||\mathcal {R}||=n$ symbols. An instance of $\textsc {GetDict}(\mathcal {R}, k)$ that uses the encoding $(H, B, H^{e})$ requires $O(|E| + u\frac{k}{w})$ words of space and runs in $O(n + (|E|-|V|)k + q)$ expected time, where *q* is the total number of times GetDict finds colliding *k*-mers in *H*.

##### Proof

The table $H^{e}$ uses $O(|E|-|V|)$ words of space as it only contains the missing edges of *G* that are not in *H*. Thus, the combined space of *H* and $H^{e}$ is *O*(|*E*|) words. If we also consider the buffer *B*, the final space usage of our compact representation is $O(|E|+u\frac{k}{w})$ words. In our new encoding, the function *keycomp* will *fully* decompress the key of a bucket $H[b']$ in one specific case: when $H[b']$ encodes *K*, but the reference $b_{pr}$ is not in $\{L,H[b'].r\}$. This situation is equivalent to discovering a new incoming edge for the node *v* labelled *label*(*K*). GetDict discovers $indegree(v)-1$ edges this way because the remaining edge is stored in $H[b'].r$ when the algorithm inserts *K* into $H[b']$. Thus, the total cost of counting the *f* occurrences of *K* in $\mathcal {R}$ (without considering collisions) is $f + (indegree(v)-1)k$. If we consider all the nodes of *G*, the total cost of counting without collisions is $O(n + (|E|-|V|)k)$ expected time. Now let us consider the collisions. The purpose of the field *d* is to ensure that we decompress a *k*-mer $K'$ from a bucket $H[b']$ only if *h* assigns the same initial bucket $h(K)=h(K')$ to *K* and $K'$. If $K=K'$, $\textsc {GetDict}$ discovered a new de Bruijn graph edge, and that cost was already covered. On the other hand, if $K \ne K'$, it means *K* and $K'$ collide. Assuming the *k*-mers collide at random in *h*, the average number of symbols to determine that two random strings do not match is constant [1]. Now, assuming GetDict found colliding *k*-mers *q* times during the scan of $\mathcal {R}$, the total cost of failed *k*-mer decompression is *O*(*q*) on expectation. This argument gives us the final $O(n + (|E|-|V|)k + q)$ expected running time. $\square$

### Dictionary of canonical *k*-mers

We present our framework to compute the *canonical* dictionary $\mathcal {D}^{c}_{k, \mathcal {R}}$. We first describe how to adapt our compact hash table to the canonical setting (“Our canonical compact hash table” section). Then, we show how to extract keys in the new encoding (“Reconstructing a *k*-mer from our canonical encoding”, and “Implementing our *k*-mer retrieval algorithm” sections), and the associated cost of the extraction (“Analysis of our *k*-mer retrieval algorithm” section). Subsequently, we present GetCanDict: our space-efficient algorithm that builds $\mathcal {D}^{c}_{k, \mathcal {R}}$ using the canonical variant of our compact hash table (“Building the canonical dictionary” section). Finally, “Correctness of our canonical encoding” section explains the correctness of the output of GetCanDict.

We remark that the canonical framework we present here does not improve the asymptotic space usage of “Building the dictionary” section, but in practice, it reduces the number of hash table entries by one-half. Additionally, the ideas we present here do not consider the improvements of “Improving the time complexity” section.

#### Our canonical compact hash table

The main difference compared to our previous scheme is that now every *k*-mer *K* occurring as a key in *H* is the canonical sequence $K^{c} \in \{K, \hat{K}\}$. This strategy collapses *K* and $K^{c}$ into one single bucket and reduces *H*’s overall size. However, it also invalidates our mechanism to spell the keys right to left as consecutive *k*-mers in $\mathcal {R}$ do not necessarily have canonical versions in the same DNA strand. In terms of the de Bruijn graph (“Connection with de Bruijn graphs” section), the reference bucket $b_{pr}$ of a *k*-mer $K^{c}$ now could be a incoming or outgoing node of $K^{c}$. This change makes the retrieval of *k*-mers from *H* more difficult compared to the non-canonical variant because our original decoding method spells $K^{c}$ by following only incoming nodes (see “Building the dictionary” section). We will adapt our technique to overcome this problem, giving a solution that keeps the advantages of the canonical encoding at the expense of performing more computations in *H*.

Our canonical encoding is closely related to the way in which our algorithm GetCanDict works (“Building the canonical dictionary” section). However, we cannot explain that algorithm without first describing the new compact encoding. For the moment, it is enough to know that, during the execution of GetCanDict, we scan the reads in $\mathcal {R}$ left to right, and for each *k*-mer $R[x\ldots y]$, we obtain its canonical sequence $K^{c}$ and insert it in some bucket *H*[*b*]. The reference bucket $b_{pr}$ we store in $H[b].r=b_{pr}$ is the one storing the canonical sequence $K^{c}_{pr}$ of $R[x-1\ldots y-1]$. When $R[x\ldots y]$ is the *k*th prefix of *R*, there is no $K^{c}_{pr}$ we can use as reference, so we store $K^{c}$ explicitly in a buffer *B*.

A relevant concept in our new scheme is that of text overlap:

##### Definition 1

*Text overlap*: let $R[x\ldots y]$ be the leftmost occurrence in $\mathcal {R}$ of $K^{c} \in \{K, \hat{K}\}$ and let $R[x-1\ldots y-1]$ be an occurrence of a string in $\{K_{pr}, \hat{K}_{pr}\}$, not necessarily the leftmost one. The text overlap of $K^{c}_{pr}$ and $K^{c}$ is the overlap between the strings of $\{K_{pr}, \hat{K}_{pr}\}$ and $\{K, \hat{K}\}$ that match $R[x-1\ldots y-1]$ and $R[x\ldots y]$, respectively. This overlap always matches a $k-1$ suffix in $\{K_{pr}, \hat{K}_{pr}\}$ with a $k-1$ prefix in $\{K, \hat{K}\}$.

**Fig. 2:**
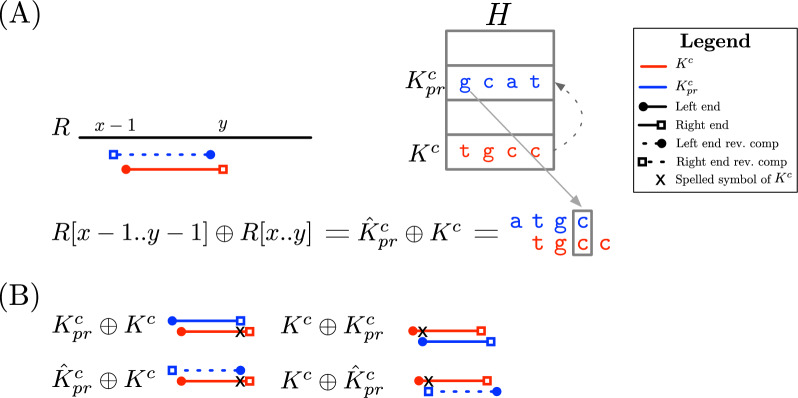
**A** Example of text overlap. The substring $R[x-1\ldots y]=\texttt {atgcc}$ encodes two *k*-mers, $R[x-1\ldots y-1] = \texttt {atgc}$ and $R[x\ldots y]=\texttt {tgcc}$. Let us assume the canonical $K^{c}_{pr} \in \{K_{pr}, \hat{K}_{pr}\}$ matches the DNA reverse complement of $R[x-1\ldots y-1]$, i.e., $\texttt {gcat}$. On the other hand, let us assume $R[x\ldots y]=K^{c}$ matches the canonical of $\{K,\hat{K}\}$. Thus, the relative DNA orientation of $R[x-1\ldots y-1] \oplus R[x\ldots y]$ with respect to $K^{c}$ is $\hat{K}^{c}_{pr} \oplus K^{c}$. This means that $K^{c}[k-1] = \pi (K^{c}_{pr}[1])=\texttt {c}$ is the symbol we obtain from the link *H*[*b*].*r* in $K^{c}$’s bucket *H*[*b*]. The grey arrow from *H* to the grey rectangle depicts this situation. **B** Text overlaps for $K^{c}_{pr}$ and $K^{c}$ relative to $K^{c}$’s DNA orientation. The *x* marks the symbol in $K^{c}$ we obtain by following *H*[*b*].*r*

The important observation about this definition is that the orientation $R[x-1\ldots y-1] \oplus R[x\ldots y]$ we encode in $H[b].r=b_{pr}$ does not necessarily match $K^{c}_{pr} \oplus K^{c}$ like in the non-canonical version of *H*. Therefore, when we set the DNA orientation of $H[b].r = b_{pr}$ with respect to $K^{c}$, *H*[*b*].*r* can spells $K^{c}[2]$ or $K^{c}[k-1]$. In general, there are four possible text overlaps for $K^{c}_{pr}$ and $K^{c}$, each with a specific DNA orientation in the spelling of $K^{c}$. Figure 2 summarises the combinations and their outcomes.

The use of text overlaps to encode the keys requires a new format for *H*. In particular, the entry $H[b]=(f,r,a,v,o, e)$ for $K^{c}$ now has six fields. The first three (*f*, *r*, *a*) have a similar meaning as in “Our compact hash table” section: *f* is the sum of the frequencies in $\mathcal {R}$ for $\{K, \hat{K}\}$, $r=b_{pr}$ is the reference bucket $H[b_{pr}]$ encoding the predecessor $K^{c}_{pr}$, and $a=K^{c}[k]$. Additionally, $v=K^{c}[1]$ is the leftmost symbol of $K^{c}$. The field *o* is a bit indicating the relative orientation of $R_{i}[x\ldots y]$ and $K^{c}$, where $R_i\in \mathcal {R}$ is the string where $K^c$ first occurred. Specifically, $o=1$ if $R_{i}[x\ldots y]=K=K^{c}$, and $o=0$ if $R_i[x\ldots y]=\hat{K} = K^{c}$. Finally, the field *e* is a bit indicating the relative orientation of $R_i[x-1\ldots y-1]$ and $K^{c}_{pr}$ with similar encoding as *o*. Notice that (*o*, *e*) encodes the text overlap of $K^{c}$ and $K^{c}_{pr}$. The next section will explain how to use the new format to recover $K^{c}$ from *H*.

#### Reconstructing a *k*-mer from our canonical encoding

Now that we have relaxed the orientation in which $K^{c}$ overlaps its predecessor $K^{c}_{pr}$, the reconstruction of $K^{c}$ might take more than *k* steps. We will slightly change the notation to explain this idea. Let $S=b_{k'},\ldots ,b_{2}, b_{1}$ be the chain of reference buckets we visit in *H* to spell $K^{c}$, with $H[b_1=b]$ storing $K^{c}$. Similarly, let $K^{c}_{k'}, \ldots , K^{c}_{1}$ be the *k*-mers these buckets represent, with $K^{c}_{1} = K^{c}$. The relationship of $K^{c}_{1}$ and $K^{c}_{2}$ is equivalent to that of $K^{c}$ and $K^{c}_{pr}$.

The general idea to reconstruct $K^{c}$ from *H*[*b*] is to scan *S* right to left, and for every bucket $H[b_j]$ we visit, we extract the symbol from one of the ends of $K^{c}_{j}$ and insert it into $K^{c}$. We refer to this procedure as *canspell*:

- *canspell*(*b*): returns the sequence $K^{c}$ from the input bucket *H*[*b*] of our compact hash table *H*.

In the non-canonical encoding of “Our compact hash table” section, we spell $K^{c}$ right to left as it holds $K^{c}_{k'} \oplus {\cdots } K^{c}_2 \oplus K^{c}_1$. In contrast, in the canonical encoding, the text overlaps of the *k*-mers in *S* do not induce a particular spelling direction. As we see in Fig. 2b, if $H[b_j].r=b_{j+1}$ represents the overlap $K^{c}_{j+1} \oplus K^{c}_{j}$, then $K^{c}_{j+1}[k]=K^{c}_{j}[k-1]$ and we retrieve a symbol from the right end of $K^{c}$. On the other hand, if $H[b_{j}].r=b_{j+1}$ represents $K^{c}_{j} \oplus K^{c}_{j+1}$, then $K^{c}_{j}[2]=K^{c}_{j+1}[1]$ and we get a symbol from the left end of $K^{c}$. Changes in the spelling direction can happen multiple times as we scan *S* and induce an *inward* reconstruction of $K^{c}$: we obtain the end symbols of $K^{c}$ and advance to its centre.

We also need to consider the orientation of the *k*-mers in *S* relative to $K^{c}$. In other words, for each $K^{c}_{j}$ in *S*, we need to know the string $K^{o}_{j} \in \{K^{c}_{j},\hat{K}^{c}_{j}\}$ overlapping $K^{c}$ according to the information in *S*. Before explaining this concept formally, we will define a function $ror_S$ that gives the relative orientation. This is its signature:

- $ror_S(j)$: returns 0 if $K^{o}_{j}=\hat{K}^{c}_{j}$, and 1 if $K^{o}_{j}=K^{c}_{j}$.

We implement $ror_S$ using the following recursive function:

1 $$\begin{aligned} ror_S(j)= {\left\{ \begin{array}{ll} 1, &{} \text {if}\ j=1 \\ \lnot ror_S(j-1), &{} \text {if}\ H[b_{j-1}].e \ne H[b_{j-1}].o\\ ror_S(j-1) &{} \text {if}\ H[b_{j-1}].e = H[b_{j-1}].o\\ \end{array}\right. } \end{aligned}$$

Initially, $ror_S(1)=1$ because the key $K^{c}_1$ in $H[b_1=b]$ is precisely $K^{c}$. Now let us assume without loss of generality that, when we visit $H[b_{j}]$ in the scan of *S*, $ror_S(j)$ returns 0. Also assume that the information in $H[b_{j}].e$ and $H[b_{j}].o$ tells us the link $H[b_{j}].r=b_{j+1}$ represents $K^{c}_{j+1} \oplus K^{c}_{j}$. We notice that the overlap $H[b_{j}].r=b_{j+1}$ is inconsistent with $ror_{S}(j)=0$ because $K^{o}_{j}=\hat{K}^{c}_{j}$ is the reverse complement of the string we use in $K^{c}_{j+1} \oplus K^{c}_{j}$. We fix this problem by flipping the DNA orientation of the overlap encoded by $H[b_j].r$ to obtain $\hat{K}^{c}_{j} \oplus \hat{K}^{c}_{j+1}$, and now the two *k*-mers are oriented with respect to $K^{c}$. However, this strand flip has a chain effect because it makes $K^{o}_{j+1}$ equal to $\hat{K}^{c}_{j+1}$ (i.e., $ror_S(j+1)=0$), and depending on $H[b_{j+1}].e$ and $H[b_{j+1}].o$, we might need to flip $H[b_{j+1}].r=b_{j+2}$ as well. We will generally continue flipping *k*-mers in *S* until we reach a bucket $b_{j'}$ where the text overlap is consistent with $ror_S(j')$.

**Fig. 3:**
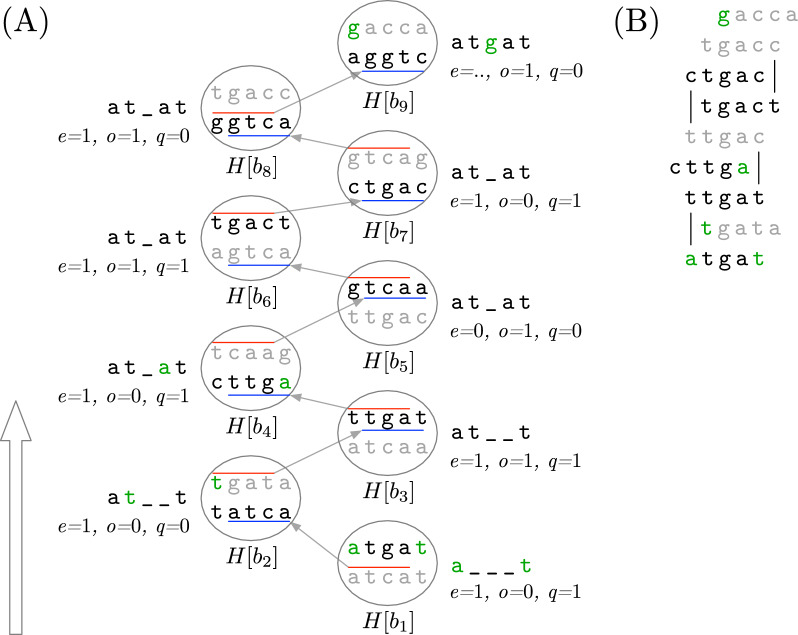
**A** Spelling $K^{c}=K^{c}_{1}=\texttt {atgat}$ from $H[b_{1}]$. The white arrow to the left indicates that the figure is read bottom-up. Each *jth* circle is the bucket $H[b_{j}]$ in the reference chain *S*. The black string is $K^{c}_{j}$ and the grey string is $\hat{K}^{c}_{j}$. The incomplete string next to each circle has the symbols of $K^{c}$ we know up to that bucket. The green symbol is the one we extract from $K^{o}_{j}$ and insert it in one of the inner ends of $K^{c}$. The arrow from $H[b_{j}]$ to $H[b_{j+1}]$ indicates the text overlap of $H[b_{j}].r = b_{j+1}$. The red line is the $k-1$ prefix in $H[b_{j}]$ that matches a $k-1$ suffix in $H[b_{j+1}]$ (blue line). **B** The *k*-mers $K^{o}_{j}$ we use in the spell of $K^c$. When we change the spelling direction from $H[b_{j+1}]$ to $H[b_{j+2}]$, $K^{o}_{j+2}$ does not match $K^{o}_{j}$ because the chain of buckets $S=b_9,\ldots ,b_2,b_1$ spelling $K^{c}$ cannot have repeated elements (see Lemma 3). We mark the mismatching symbols of $K^{o}_{j}$ and $K^{o}_{j+2}$ with vertical lines in the figure. On the other hand, we remark that changes in the spelling direction are induced by the order in which we insert the *k*-mers in *H* and the reference bucket we have available at the moment of the insertion

Considering all this information, we can fairly say that traversing *S* right to left resembles sliding a window over $K^{c}$ back and forward as we visit the buckets. Initially, the window is set to $w_{\ell }=1, w_r=k$ when we are in $H[b_1=b]$. Then, when we reach $H[b_{j}]$, we move the window to the left: $w_{\ell } = w_{\ell }-1, w_r=w_r-1$ if $K^{o}_{j+1} \oplus K^{o}_{j}$ is the orientation of $H[b_{j}].r=b_{j+1}$ relative to $K^{c}$. In contrast, we move the window to the right: $w_{\ell }= w_{\ell }+1, w_r = w_r+1$ if $K^{o}_{j} \oplus K^{o}_{j+1}$ is the orientation of $H[b_{j}].r=b_{j+1}$ relative to $K^{c}$. Figure 3b shows an example of this idea.

The implementation of *canspell*(*b*) thus translates to extracting symbols from the distinct *k*-mers $K^{o}_{j}$ we visit as we slide $w_{\ell }, w_r$, stopping only when we have covered all the positions of $K^{c}$. As mentioned before, this mechanism reconstructs $K^{c}$ inwards.

We will keep two variables $c_{\ell }=1,c_r=k$ that mark the inner ends of the reconstruction. Initially, the symbols within $K^{c}[c_\ell \ldots c_r]$ are unknown, but we will obtain them as we slide $w_{\ell }, w_r$. When we recover $K^{c}[c_{\ell }]$, we update the inner left end $c_{\ell }=c_{\ell }+1$, and when we obtain $K[c_r]$, we update the inner right end $c_{r}=c_r-1$. Notice that *canspell* will run as long as $c_{\ell }\le c_{r}$.

Our canonical encoding only enables the extraction of the symbols $K^{o}_{j}[1]$ and $K^{o}_j[k]$ of each *k*-mer $K^{o}_{j}$ (fields *a* and *v* in *H*), meaning that we cannot recover $K^{c}[c_{\ell }]$ or $K^{c}[c_r]$ every time we visit a bucket in *S*. We consider the following two scenarios to extract symbols:

1. $w_{\ell }<1$, $1 \le w_r < k$: if $c_r=w_r$, then it holds $K^{o}_{j}[k-w_{r}+1\ldots k] = K^{c}[1\ldots w_{r}=c_r]$, so we recover $K^{c}[c_r]=K^{o}_{j}[k]$.
2. $w_r>k$, $1<w_{\ell } \le k$: if $c_{\ell }=w_{\ell }$, then it holds $K^{c}[c_{\ell } = w_{\ell }\ldots k] = K^{o}_{j}[1\ldots k-w_{\ell }+1]$, so we recover $K^{c}[c_{\ell }]=K^{o}_{j}[1]$.

It is also worth mentioning that when we reach a bucket $b_{j}$ in *S* whose key is in *B*, we have direct access to the full sequence of $K^{o}_{j}$. Therefore, we copy the corresponding area of $K^{o}_{j}$ within $K^{c}[c_{\ell }\ldots c_r]$ and finish *canspell*(*b*). This condition makes $b_{j}$ the last bucket in *S*.

We now show that *canspell* always returns an answer and that that answer is the correct sequence of $K^{c}$.

##### Lemma 3

The execution of *canspell*(*b*) always finishes and returns a *k*-mer.

##### Proof

We first demonstrate that *S* does not have loops $b_{j}, b_{j+x},\ldots , b_{j+1}, b_{j}$. This type of structure would produce that, after visiting $b_{j+x}$, we return to $b_{j}$ and thus never end the reconstruction of $K^{c}$. GetCanDict fills *H* keeping the following invariant: when we insert $K^{c}_{j}$ in $b_{j}$, the bucket $H[b_{j+1}]$ already encodes $K^{c}_{j+1}$ and $H[b_{j}]$ is empty, so it is safe to store $H[b_{j}].r=b_{j+1}$. The invariant, in turn, induces the transitive property that all the buckets $b_{j}, b_{j+x},\ldots , b_{j+1}$ of *S* already contained a *k*-mer when we inserted $K^{c}_{j}$. Further, $H[b_{j}]$ was empty and $K^{c}_{j}$ did not exist as a key up to that point. However, the loop $b_{j}, b_{j+x},\ldots , b_{j+1}, b_{j}$ contradicts these ideas because the leftmost occurrence of $b_{j}$ indicates that $H[b_{j}]$ already contained $K^{c}_{j}$ when we inserted it and created the link $H[b_{j}].r=b_{j+1}$ (rightmost occurrence of $b_{j}$ in the loop). Thus, we conclude that *S* cannot have a loop. $\square$

We remark that the absence of cycles in *S* (Lemma 3) implies that not all the *k*-mers encoded by *S* overlap $K^{c}$. Suppose that, starting from $K^{o}_{j}$, we slide the window *x* positions to the right and then $x'<x$ positions to the left. When we return the window back to the left, the *k*-mers we visit are not the same as those we visited when sliding the window to the right, otherwise it would mean *S* has a cycle. If we combine this idea with the fact that the *k*-mers in *S* have transitive overlaps, we have that $K^{o}_{j}$ does not have a suffix-prefix overlap with $K^{o}_{j+x+x'}$, but a substring match (see the vertical lines in Fig. 3b). However, this condition does not prevent us from reconstructing $K^{c}$.

##### Lemma 4

*canspell*(*b*) returns the canonical *k*-mer $K^{c}$ encoded by the bucket *H*[*b*].

##### Proof

We show that each *k*-mer $K^{o}_{j}$ we obtain from *S* has a substring matching $K^{c}[c_\ell \ldots c_{r}]$ at the moment we reach the bucket $b_{j}$. We refer to this idea as the *matching* property. The validity of the matching property proves *canspell* outputs $K^{c}$ correctly because the algorithm obtains $K^{c}[c_\ell ]$ or $K^{c}[c_r]$ from the substring of $K^{o}_j$ matching $K^{o}[c_{\ell }\ldots c_r]$. Our proof below uses an arbitrary sequence of slides for $w_\ell , w_r$, but we can give a symmetric argument for other sliding sequences.

When we start *canspell*(*b*), the matching property is trivially true as $c_{\ell }=1, c_{r}=k$ and the bucket $H[b_1=b]$ encodes precisely the *k*-mer $K^{o}_{1} = K^{c}$. Now assume the sequence $b_{j},b_{j-1},\ldots b_{1}$ in *S* slide the window $j-1<k$ positions to the left, so the right boundary now is $c_{r}=c_r-j+1$. The matching property still holds as the *k*-mers $K^{o}_{j}, K^{o}_{j-1}, \ldots , K^{o}_{2}$ have a suffix overlapping the prefix $K^{c}[1\ldots c_{r}=k-x+1]$, with $x \in [2\ldots j]$, due to the transitive overlaps $K^{o}_{j} \oplus K^{o}_{j-1}{\cdots }K^{c}$. The active area we need to compute becomes $K^{o}_{j}[j\ldots k-1] = K^{c}[c_\ell =1\ldots c_r=k-j]$ as we moved $c_r$ inwards after processing $H[b_{j}]$. Now assume that the next *u* buckets in *S* slide the window *u* positions to the right (i.e., we change the sliding direction). We distinguish three cases:

- $u<j-1$: we know that $K^{o}_j[u+1\ldots k] = K^{o}_{j+u}[1\ldots k-u]$ holds because of the transitive overlaps $K^{o}_{j} \oplus K^{o}_{j+1}{\cdots }K^{o}_{j+u}$ when sliding the window to the right. If we consider this match and $K^{o}_{j}[j\ldots k-1]=K^{c}[c_{\ell }\ldots c_{r}]$, then by the transitive overlaps $K^{o}_{j} \oplus K^{o}_{j+1} {\cdots } K^{o}_{j+u}$ when sliding the window to the right, we get the match $K^{o}_{j+u}[j-u\ldots k-1-u]=K^{c}[c_{\ell }\ldots c_r]$. Notice that $K^{o}_{j+u}[j-u\ldots k-1-u]$ is not a prefix or a suffix because $j-u>1$ and $k-1-u<k$, and our encoding does not support direct access to this area of $K^{o}_{j+u}$. This situation means that we cannot shrink $K^{c}[c_{\ell }\ldots c_r]$ as we visit $K^{o}_{j+u}$, or any in any of the *k*-mers $K_{j+u-1}, \ldots , K^{o}_{j+1}$.
- $u=j-1$: the match $K^{o}_{j+u}[j-u\ldots k-1-u]=K^{c}[c_{\ell }\ldots c_r]$ becomes $K^{o}_{j+u}[1\ldots k-j]=K^{c}[c_{\ell }\ldots c_r]$. $K^{o}_{j+u}$ and $K^{c}$ have the same sliding window position $w_{\ell }=1, w_r=k$, but they have different sequences due to Lemma 3. However, the substring of $K^{o}_{j+u}$ matching $K^{c}[c_\ell \ldots c_r]$ is a prefix and we have access to $K^{o}_{j+u}[1]$ in our encoding. Therefore, we extract $K^{c}[c_{\ell }]$ and move $c_{\ell }$ one position inwards $c_{\ell }=c_{\ell }+1$.
- $j\le u$: for the buckets $b_{j+1},\ldots , b_{j+j-1}$ the previous cases apply. The remaining buckets $b_{2j},\ldots , b_{j+u}$ move the inner left end $c_{\ell }$ by one position each because the matches induced by the transitive overlaps $K^{o}_{2j} \oplus K^{o}_{2j+1}{\cdots } K^{o}_{j+u}$ when sliding the window to the right go in the same direction as we move $c_{\ell }$. For every $K^{o}_{x}$, with $x \in [2j\ldots j+u]$, we have $K^{o}_{x}[1\ldots c_r-c_{\ell }+1] = K^{c}[c_{\ell }\ldots c_r]$, and because we have access to $K^{o}_{x}[1]$ in our encoding, we can retrieve $K^{c}[c_{\ell }]$ and move $c_{\ell }=c_{\ell }+1$. Thus, the inner left end becomes $c_{\ell }=c_{\ell } + u-j+1$ after visiting $b_{j+u}$.

After consuming $b_{j+u},\ldots ,b_{2}$, it might happen that the window changes direction again to the left. However, in this scenario, symmetrical conditions to those explained above apply. $\square$

We will present a formal implementation of *canspell* in the next section.

#### Implementing our *k*-mer retrieval algorithm

This section describes the practical aspects of implementing $canspell(b)=K^{c}$. We present the pseudocode in Algorithm 2 and explain all the details below.

We begin (Lines 1–3) by initialising the variables $c_{\ell }=1,c_r=k$ that mark the inner left and right ends of $K^{c}$ (respectively) in the inward reconstruction. We also define a bit $q=ror_{S}(j)$ that tells us the string $K^{o}_{j} \in \{K^{c}_{j}, \hat{K}^{c}_{j}\}$ overlapping $K^{c}$. We set the initial value $q=ror_{S}(1)=1$ according to Eq. 1.

We continue *canspell* by traversing *S* right to left. Every time we reach a new bucket $H[b_j]$, we perform three steps:

(i) Check if the key of $H[b_{j}]$ is in the buffer *B*.
(ii) Check if the window $w_{\ell }, w_r$ crosses the inner ends of $K^{c}[c_{\ell }\ldots c_{r}]$.
(iii) Slide the window in some direction and move to the next bucket $H[b_{j+1}]$ in *S*.

Lines 5–13 show the process of step (i). Recall from “Our canonical compact hash table” section that we store *k*-mers explicitly in a dynamic buffer *B* whenever we do not have a predecessor we can reference in *H*, flagging the buckets in this situation with $\varepsilon$. Thus, when the traversal of *S* reaches a bucket $b_{j}$ such that $H[b_{j}].r=\varepsilon$, it means the full sequence of $K^{c}_{j}$ is in $B[l\ldots l+k-1]$, with $l=H[b_{j}].r$. The advantage of the buffer is that it contains all the information we need to complete the inner substring $K^{c}[c_{\ell }\ldots c_r]$ (see Lemma 4). Therefore, we get the positions $o_{\ell }=c_{\ell }-w_{\ell }+1, o_{r}=o_{\ell }+c_r-c_{\ell }$ of the substring $K^{o}_{j}[o_{\ell }\ldots o_{r}] = K^{c}[c_{r}\ldots c_{\ell }]$. Subsequently, if $K^{o}_{j}$ matches $K^{c}_{j}$ ($q=1$), we copy $B[l+o_{\ell }-1\ldots l+o_{r}-1]$ in $K^{c}[c_{\ell }\ldots c_r]$. In contrast, when $K^{o}_{j}$ matches $\hat{K}^{c}_{j}$ ($q=0$), we copy the reverse complement of $B[l+k-o_r\ldots l+k-o_{\ell }]$ in $K^{c}[c_{\ell }\ldots c_r]$ instead. We finish the execution of *canspell* by returning $K^{c}$. On the other hand, if the key of $H[b_j]$ is not in *B*, we move to step (ii).

Lines 14–19 represent the work of step (ii). We start by computing the ends of $K^{o}_{j}$ using the bit *q*. Specifically, if $q=ror_{S}(j)=1$, we get $K^{o}_{j}[1]=K^{c}_{j}[1]=H[b_j].v$ and $K^{o}_{j}[k] = K^{c}_{j}[k] = H[b_{j}].a$. In contrast, when $q=ror_{S}(j)=0$, we get $K^{o}_{j}[1]=\pi (K^{c}_{j}[k]) = \pi (H[b_{j}].a)$ and $K^{o}_{c}[k]=\pi (K^{c}_{j}[1])=\pi (H[b_{j}].v)$. We continue by checking if the window $w_{\ell }, w_r$ crosses an inner end of $K^{c}$. If that is the case, we insert $K^{o}_{j}[1]$ or $K^{o}_{j}[k]$ in $K^{c}$ depending on which matching scenario holds (see Cases 1 and 2 at the end of “Reconstructing a *k*-mer from our canonical encoding” section).

Lines 20–30 depict the work we perform during step (iii). We first infer the direction (left or right) in which we slide the window. For that purpose, we rely on the text overlaps we presented in Fig. 2b. Recall that our encoding sets $H[b_{j}].o=1$ if the link $H[b_{j}].r=b_{j+1}$ uses $K^{c}_{j}$ for the text overlap between $K^{c}_{j+1}$ and $K^{c}_{j}$, and 0 if it uses $\hat{K}^{c}_{j}$. Equivalently, $H[b_{j}].e=1$ means the link uses $K^{c}_{j+1}$ for the overlap, and $H[b_{j}].e=0$ means $\hat{K}^{c}_{j+1}$. Thus, we slide the window to the left if $H[b_{j}].o=1$, and to the right if $H[b_{j}].o=0$.

When computing the sliding direction, we also need to consider the orientation of $K^{c}_{j}$ relative to $K^{c}$. In particular, if $ror_{S}(j)=0$, we invert the slide direction, otherwise we leave it unchanged. It is not difficult to see why we need to do this inversion: suppose $H[b_j].r=b_{j+1}$ defines the overlap $K^{c}_{j+1} \oplus K^{c}_{j}$ but $q=ror_{S}(j)$. The overlap indicates that we need to slide the window to the left, but the bit in *q* indicates we need to flip the overlap to $\hat{K}^{c}_{j} \oplus \hat{K}^{c}_{j+1}$, which is equivalent to sliding the window to the right.

After setting the slide direction, we update *q* as follows: if $H[b_{j}].e \ne H[b_{j}].o$, we flip $q=\lnot q=ror_{S}(j+1)$, otherwise we leave it unchanged (i.e., $q=ror_S(j)=ror_S(j+1)$). Finally, we move to the next bucket $H[b_{j+1}]$ and repeat the same three steps above.

The reconstruction of $K^{c}$ finishes when $c_{\ell }>c_r$, which means we already cover all the symbols of *H*[*b*]’s key. Figure 3 and Example 1 show how we reconstruct $K^{c}$ in the canonical encoding.

**Algorithm 2:**
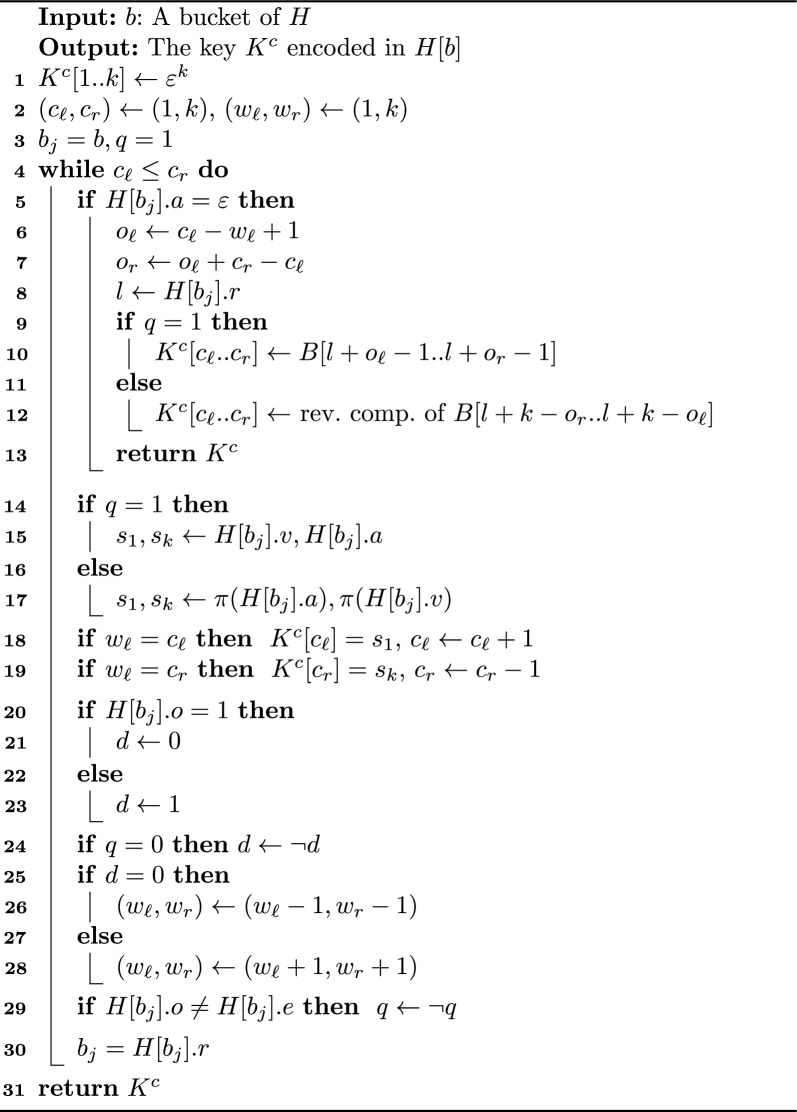
Pseudocode of *canspell*

##### Example 1

Spelling of $K^{c}=K^{c}_{1} = \texttt {atgat}$ from $H[b_{1}]$ in Fig. 3. We initialise the sliding window $w_{\ell }=1,w_r=k$ and the inner ends $c_{\ell }=2,c_{r}=4$ of $K^{c}$ as $H[b_{1}]$ already stores $K^{c}[1]$ and $K^{c}[k]$. Then, the fields $e=1,o=0$ in $H[b_1]$ indicate the overlap $K^{c}_{1} \oplus \hat{K}^{c}_{2}$, which means sliding the window to the right $w_{\ell }=2, w_r=6$. Besides, as $q=ror_S(1)=1$, we do not invert the sliding direction. However, as $H[b_1].e\ne H[b_1].o$, *q* becomes $ror_S(2)=\lnot ror_S(1)=0$. When we visit $H[b_2]$, $w_{\ell }$ matches $c_{\ell }=2$, so we set $K^{c}[c_{\ell }=2]=K^{o}_{2}[1]=\pi (K^{c}_{2}[k])$ and move $K^{c}$’s inner left end to $c_{\ell }=3$. The fields $e=1,o=0$ in $H[b_{2}]$ indicate $K^{c}_{2} \oplus \hat{K}^{c}_{3}$, which means sliding the window to the right, but as $K^{o}_2=\hat{K}^{c}_{2}$ ($q=0$), we invert the direction and slide the window to the left $w_{\ell }=1, w_r=5$ instead. Further, $q=\lnot ror_S(2)=ror_S(3)$ becomes 1 as $H[b_{2}].e \ne H[b_{2}].o$. When we visit $H[b_3]$, the window does not match the inner ends $c_{\ell }=3,c_r=4$, so we do not recover any symbol. Additionally, $H[b_3].e=1$ and $H[b_3].o=1$ indicate $K^{c}_{4} \oplus K^{c}_{3}$, and because $q=1$, we slide the window to the left $w_{\ell }=0, w_{r}=4$. The bit $q=ror(3)=ror(4)$ remains the same as $H[b_3].e=H[b_3].o$. In $H[b_{4}]$, it holds $c_r=w_r=4$, so we get $K^{c}[c_r=4]=K^{o}_{4}[5]$ and set $c_r=3$. The window does not match the inner ends $c_{\ell }=3, c_r=3$ in buckets $b_5,b_6,b_7$, and $b_{8}$, so we do not get any symbol. The window in $H[b_{9}]$ is $w_{\ell }=3, w_r=7$, and because $w_{\ell }=c_{\ell }=3$, we get $K^{c}[c_{\ell }=3]=K^{o}_{9}[1]=\pi (K^{c}_{9}[k])$, set $c_{\ell }=4>c_r=3$, and we are done.

#### Analysis of our *k*-mer retrieval algorithm

We now analyse the cost of running *canspell*. We will see that its execution is exponential in the worst case, but our experiments showed that it is much faster in practice (see Fig. 9). We summarise the running time of *canspell* with this theorem:

##### Theorem 5

The function $canspell(b)=K^{c}$ returns in $O(\sigma ^{k})$ time the canonical sequence $K^{c}$ stored in the bucket *H*[*b*] of our canonical compact hash table *H*.

##### Proof

Since all buckets in *S* are distinct, they represent different canonical *k*-mers. There are at most $O(\sigma ^k)$ different canonical *k*-mers and thus *canspell*(*b*) visits at most $O(\sigma ^k)$ buckets. Processing each bucket requires constant time, and thus, the function *canspell*(*b*) has time complexity $O(\sigma ^{k})$. $\square$

Our proof of Theorem 5 demonstrates in a simple way that the running time of *canspell* is exponential in the worst case. However, the proof is incompatible with our proof of Lemma 4, which states that all the *k*-mers $K^{o}_{j}$ encoded by *S* share a substring with $K^{c}$. In the following, we will present an analysis that shows that we have an exponential upper bound for our *k*-mer retrieval algorithm even if all the $K^{o}_{j}$ in *S* have a match with $K^{c}$.

We will assume *canspell* only extends the inner left end $c_{\ell }$ of $K^{c}$ as it traverses the buckets of *S*, and maintains the invariant that $c_r = k$. This assumption is only to simplify our explanations, and it does not affect the correctness of the reconstruction.

Let *o* be the number of symbols in $K^{c}$ we have already discovered at some point in the execution of *canspell*. As we fixed $c_{r}=k$, it holds $o=c_{\ell }$. We will prove our theoretical bound by studying, for each value of $o\le k$, the maximum number of buckets we could visit in *S* before moving $c_{\ell }$ one position. We finish the construction of $K^{c}$ after moving $c_{\ell }$ *k* times.

When $o=0$, the cost of moving $c_{\ell }$ is *O*(1) as the first bucket $b_{1}$ in *S* is precisely the one encoding $K^{c}$, and our compact encoding *H* keeps $K^{c}[1]$ in $H[b_1]$.

**Fig. 4:**
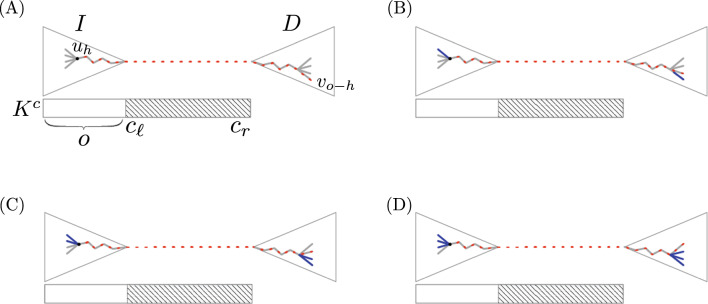
Demonstration of the running time of *canspell*. **A** The current bucket $b_{j}$ in *S*’s traversal stores $K^{c}_{j}=label_{I}(u_{h}){\cdot }K^{c}[c_{\ell }\ldots c_r]{\cdot }label_D(v_{o-h})$ (dotted red line). The triangles are the tries *I* and *D*. **B** Sliding $w_{\ell }, w_r$ $x=1$ position to the left, and then $x=1$ position to the right (i.e., visiting the next two buckets in *S*). The new node $v_{o-h}$ is different from that of **A** because of Lemma 3. We already traversed the blue path and can not traverse it again due to Lemma 3. **C**, **D** The remaining window sliding options visiting $u_{h}$. After (**D**), we can only slide $w_{\ell }, w_r$ to the right without breaking Lemma 3. Therefore, we visited $u_{h}$ $\sigma -1=3$ times, with $\sigma$ being the trie’s degree

Now we study the case $0<o\le k$. Figure 4 depicts a graphical example of our argument. We consider two copies of the trie encoding the strings in $\Sigma ^o$. We will refer to them as *I* and *D*, and will associate *I* with moving $w_{\ell },w_{r}$ to the left and *D* with moving $w_{\ell },w_r$ to the right. Let $u_h$ be a node at height *h* in any of these tries. We will use the notation $u_{h}, u_{h+1}$ to indicate we descend from $u_{h}$ to its child $u_{h+1}$, and $u_{h+1}, u_h$ to indicate we climb from $u_{h+1}$ to its parent $u_{h}$.

Let $u_h \in I$ be a node at height *h* and let $v_{o-h} \in D$ be a node at height $o-h$. The operator $label_I(u_h)$ returns the string spelled by the path $u_h,u_{h-1}, \ldots , u_1$ from $u_{h}$ to the root $u_{1}$ of *I*, while $label_D(v)$ is the string spelled by the path $v_{1},v_2, \ldots , v_{o-h}$ starting at the root $v_{1}$ of *D* and ending at $v_{o-h}$. The string $label_{I}(u_{h}){\cdot }K^{c}[c_{\ell }\ldots c_r]{\cdot }label_D(v_{o-h})$ forms one of the *k*-mers encoded by *S* before moving the inner end $c_{\ell }$. Although different nodes $u_o \in I$ and $v_{o-h} \in D$ form different *k*-mers, Lemma 3 limits the node combinations that form *k*-mers we could see in *S*. We will explain this idea below.

Moving $w_{\ell }, w_r$ to the left by $x \le o-h$ positions translates to descend a path $u_{h+1}, u_{h+2}, \ldots , u_{h+x}$ in the subtree of *I* rooted at $u_{h}$, at the same time it means climbing the *x* ancestors $v_{o-h-1}, v_{o-h-2},\ldots , v_{o-h-x}$ of $v_{o-h}$ in *D*. Moving the window to the right represents the opposite operation: climbing the ancestors of $u_{h+x}$ and descending a path from the subtree of *D* rooted at $v_{o-h-x}$. However, when we move to the right, we cannot descend the same path $v_{o-h-x+1}, v_{o-h-x+2}, \ldots , v_{o-h}$ we climbed in *D* before because it would mean visiting *k*-mers of *S* more than once, which contradicts Lemma 3. Symmetrically, once we choose a path in *D* to move to the right, if we require to move to the left again, we can not descend $u_{h+1}, u_{h+2}, \ldots , u_{h+x}$ in *I*.

In conclusion, sliding $w_{\ell },w_r$ back and forward over a node in *I* or *D* cancels branches, reducing the formation of *k*-mers that we could see in *S*. Figure 3B depicts this situation with vertical lines. The cancellations produce each internal node *v* in *I* or *D* to be associated with $\sigma -1$ different *k*-mers in *S* as we can descend only once through each of *v*’s children and then climb back to *v*. We use the remaining child to slide the window in the same direction we did before, thus keeping the invariant of Lemma 3. On the other hand, every leaf in the tries is associated with one *k*-mer of *S*. Once we exhausted all the possible visits in all the nodes in *I* and *D*, we do not have any other choice but to slide $w_{\ell }, w_r$ to the right and move $c_{\ell }$.

The trie with the strings in $\Sigma ^{o}$ is a full *k*-ary tree of degree $\sigma$, meaning it has $\sigma ^{o}$ leaves and $\sigma ^{o}-1/ \sigma -1$ internal nodes. If we add the cost of the $\sigma -1$ possible visits to an internal node, we obtain a cost of $\sigma ^{o}-1$ for processing the internal nodes and a total cost of $2\sigma ^{o}-1$ for processing the full trie.

Now let us assume we moved the inner end $c_{\ell }$ one position, so now we know $o+1$ symbols of $K^{c}$. Let $I^{o}, D^{o}$ be the tries we used for *o* and let $I^{o+1},D^{o+1}$ be the new tries we will use for $o+1$. We know that $I^{o}$ is a subtree of $I^{o+1}$ (respectively, $D^{o}$ of $D^{i+1}$) that we already traversed when the number of known symbols of $K^{c}$ was *o*. Therefore, if at some point in the synchronized traversal of $I^{o+1}$ and $D^{o+1}$, we visit two nodes $u_{h}$ and $v_{o+1-h}$ such that $u_{h}$ belongs to the subtree $I^{o}$ and $v_{o+1-h}$ belongs to $D^{o}$, then we would form a *k*-mer we already visited, thus breaking Lemma 3. Therefore, the cost of moving $c_{\ell }$ when $o+1$ becomes $(2\sigma ^{o+1}-1) - (2\sigma ^{o}-1)$ as we discard the subtrees $I^{o}$ and $D^{o}$ of visited *k*-mers. Further, if we consider all the possible values for *o*, we obtain

2 $$\begin{aligned} \sum ^{k}_{o=1} (2\sigma ^{o}-1) - (2\sigma ^{o-1}-1) \end{aligned}$$

Equation 2 is a sum that telescopes to $2\sigma ^{k}-1$, so we obtain the running time $O(\sigma ^{k})$ for *canspell*.

The analysis we presented here is rather pessimistic as having full tries *I* and *D* implies that the input read collection $\mathcal {R}$ produces a complete de Bruijn graph with order *k*, which is hardly the case in practical scenarios.

#### Building the canonical dictionary

Our algorithm GetCanDict constructs the canonical dictionary $\mathcal {D}^{c}_{\mathcal {R}, k}$ using our compact data structure of “Our canonical compact hash table” section. The idea is to scan $\mathcal {R}$ left to right, and for each *k*-mer $K=R[x\ldots y]$, we compute its canonical $K^{c} \in \{K, \hat{K}\}$ and insert $K^{c}$ into *H* using the operation *incval* (see “Building the dictionary” section). The most relevant change of GetCanDict compared to GetDict is the implementation of $keycomp(H, b', K^{c}, b_{pr})$, the subroutine that *incval* calls to compare *k*-mers while probing buckets in the compact hash table (see Algorithm 4.2.2).

The function $keycomp(H, b', K^{c}, b_{pr})$ receives a bucket $H[b']$ and an input *k*-mer $K^{c}$, and returns *true* if $K^{c}$ matches the key in $H[b']$, and *false* otherwise. The parameter $b_{pr}$ is a bucket $H[b_{pr}]$ encoding $K^{c}_{pr}$. In the canonical variant of *keycomp*, we will add two extra parameters $e'$ and $o'$. Let $R[x\ldots y]$ be the substring in $\mathcal {R}$ where we obtain the occurrence of $K^{c}$ we are querying in *keycomp*, and let $R[x-1\ldots y-1]$ the occurrence of the predecessor $K^{c}_{pr}$ associated with $H[b_{pr}]$. The field $o'$ tells the relative orientation of $K^{c}_{pr}$ and $R[x-1\ldots y-1]$ and the field $e'$ tells the relative orientation of $K^{c}$ and $R[x\ldots y]$. The meaning of the values for $e'$ and $o'$ is the same as those for $H[b'].e$ and $H[b'].o$.

We start the canonical variant of *keycomp* by comparing the tuple $(K^{c}[1],K^{c}[k], b_{pr}, e', o')$ against ($H[b'].v$, $H[b'].a$, $H[b'].r$, $H[b'].e$, $H[b'].o$). If they are equal, we conclude that $K^c$ equals the *k*-mer in $H[b']$, so we return *true*. If they differ, we need to reconstruct $H[b']$’s key to check if it matches $K^c$.

This process is almost the same as running *canspell* on $H[b']$ (Algorithm 2). The only difference is that every time we extract symbols for $H[b']$’s key (Lines 10, 12,18, or 19), we compare them against their corresponding positions in $K^{c}$, and if they differ, we return *false*. If all the symbols in $H[b']$’s key matched their corresponding positions in $K^{c}$, we return *true*.

We also introduce a small modification to *incval* to maintain the correctness of the dictionary. In the non-canonical scheme, when we call *incval* with a *k*-mer *K* that currently is in *B*, but now we have a predecessor bucket $b_{pr}$ for it, we remove *K* from *B* and update the predecessor reference in $H[b].r=b_{pr}$ (see Line 24 in Algorithm 4.2.2). In the canonical variant of *incval*, we do something similar: we remove a canonical sequence $K^{c}$ from *B* if we now know a bucket $b_{pr}$ we can use for its reconstruction. However, we also need to ensure that all *k*-mers in *H* can be reconstructed after this update. This invariant can be violated if the reconstruction of the key $K^{c}_{pr}$ in $H[b_{pr}]$ depends on the reconstruction of $K^{c}$. In this situation, setting $H[b].r=b_{pr}$ creates a cycle in the chain *S* spelling $K^{c}_{pr}$ from $H[b_{pr}]$, thus invalidating Lemma 3. We can easily check this condition by calling *keycomp* on bucket $b_{pr}$ and checking if the chain of references includes $K^c$. If this is the case, we keep $K^{c}$ in *B* to ensure that all *k*-mers in *H* are reconstructible.

#### Correctness of our canonical encoding

We will show that the canonical encoding of *H* and the way GetCanDict works do not prevent the correct construction of the *k*-mers in $\mathcal {D}^{c}_{k, \mathcal {R}}$. The only important aspect to demonstrate is that *keycomp* always returns the correct answer.

##### Lemma 6

Let $K^{c} \in \{K, \hat{K}\}$ be a canonical *k*-mer encoded in *H*[*b*] with an occurrence $K=R_{i}[x\ldots y] \in \mathcal {R}$. Additionally, let $H[b_{pr}]$ be the bucket encoding the canonical form of $K_{pr}=R_{i}[x-1\ldots y-1]$. Given an arbitrary non-empty bucket $H[b']$, $keycomp(H, b', K^{c}, b_{pr}, e', o')$ will always stop and return *true* or *false*.

##### Proof

At the beginning of GetCanDict, *H* is empty and thus trivially all *k*-mers can be reconstructed. Let us assume that before we insert a new *k*-mer $K^{c}$ into *H*, all *k*-mers already in *H* can be reconstructed. When inserting $K^{c}$, we will either (i) add $K^{c}$ to the dynamic buffer *B* and store a pointer for $B[l\ldots l+k-1]=K^{c}$ in $H[b].r=l$ or (ii) add the first and last symbols of $K^{c}$ to *H*[*b*] together with the bucket $H[b].r = b_{pr}$ of $K^{c}_{pr}$. In case (i), $K^{c}$ clearly can be reconstructed as we only need to access *B* using $H[b].r=l$. In case (ii), we immediately know the first and the last symbols of $K^{c}$. Furthermore, we have the bucket $H[b_{pr}]$ storing the *k*-mer $K^{c}_{pr}$. By definition, we know that $K^{c}$ overlaps by $k-1$ symbol one of the strings $\{K_{pr}, \hat{K}_{pr}\}$ from which $K^{c}_{pr}$ was obtained (bits *H*[*b*].*o* and *H*[*b*].*e* tell us which string, $K_{pr}$ or $\hat{K}_{pr}$, is the one that $K^{c}$ overlaps). Since $K^{c}_{pr}$ is already in *H*, it can be reconstructed, and thus we can uncover the remaining $k-2$ symbols of $K^{c}$. $\square$

### Reporting the *k*-mers in the compact dictionary

Our framework also implements an algorithm to report the *k*-mers of a compact hash table *H* in uncompressed form. We refer to this procedure as DumpDict. It works by following the reference chains to reconstruct the *k*-mers and write them into an output file along with their frequencies, provided the frequency is above some input threshold $\tau$. “Non-canonical scheme” section explains how DumpDict works when *H* follows the non-canonical scheme, and “Canonical scheme” section describes how it works when *H* follows the canonical scheme.

#### Non-canonical scheme

In the non-canonical version of *H*, the process is simple: we scan *H* left to right until we find the leftmost occupied bucket *H*[*i*]. Let $S_i=b_{k'},\ldots ,b_2,b_1$ be the reference chain starting at $H[i=b_1]$, with $H[b_{j}].r=b_{j+1}$ and $H[b_{k'}]$ being the only bucket that does not have a reference (i.e., $K_{k'}$ is the dynamic buffer *B*). We also assume that $S_i$ has length $|S_i|=k'\ge k$, meaning that the chain encodes one or more *k*-mers together. We take the symbol in $H[b_{1}].a$ and store it in the rightmost position *W*[*k*] of a buffer $W[1\ldots k]$. Then, we follow the link $H[b_1].r=b_{2}$, retrieve the DNA symbol in $H[b_{2}]$, and add it to $W[k-1]$. We keep applying this idea until we have *k* symbols in *W*. Notice we fill *W* from right to left because the DNA symbols we store in *H* are in the right end of their *k*-mers. Once *W* is full, we are ready to print the *k*-mer $K_{1}$, the one stored in $H[i=b_{1}]$. We find its count in *H*[*i*].*f*, and if it is above $\tau$, we write *W* and its frequency in the output file. Then, we follow the reference chain further $H[b_{k}].r=b_{k+1}$. Since *W* is full, we have to drop the rightmost symbol *W*[*k*], shift the buffer by one position to the right and insert the DNA symbol of $H[b_{k+1}]$ in *W*[1] to get $K_2$. We then check its frequency in *H*[*H*[*i*].*r*] and decide whether to print it or not using $\tau$. We continue traversing *S*_*i*_ until reaching $K_{k'}$, or until we encounter a *k*-mer that has already been printed. To do this, when a bucket is visited, we mark it so we do not later try to print the same *k*-mer again. After we process all the *k*-mers in $S_{i}$, we move to the next occupied bucket $H[i']$, with $i'>i$, in the scan of *H*, and start to process the corresponding reference chain $S_{i'}$, unless $H[i']$ is marked as processed. In this case, we move to the next occupied bucket and continue with the same idea until we finish the scan of *H*.

We need the *k*-mer reference chains to be as long as possible to make the writing process more efficient. The reason is that we need to visit *k* buckets to reconstruct the first *k*-mer in a reference chain $S_{i}$, but all subsequent *k*-mers in $S_{i}$ can be reconstructed by visiting one new bucket. So, instead of starting the writing process at the leftmost occupied bucket *H*[*i*], we can scan *H* once and mark all buckets that are referenced by another *k*-mer. Now, all buckets that are not marked are either empty or contain a *k*-mer that is not referenced by another *k*-mer. We can then start writing *k*-mers from these unmarked buckets to maximise the reference chain lengths and reduce the time needed to print the *k*-mers.

#### Canonical scheme

The *k*-mer writing process in the canonical variant of *H* is more involved but follows the same idea we described in “Non-canonical scheme” section. The first *k*-mer $K^{c}_1$ in a reference chain $S_i=b_{k'},\ldots ,b_2,b_1$ is fully reconstructed by calling $W=canspell(b_1)$ (Algorithm 2). Let us assume this instance of *canspell* used the leftmost $k\le j\le k'$ buckets $b_{j},\ldots , b_2, b_1$ of $S_i$ to get $K^{c}_{1}$. Then, reconstructing the next *k*-mer $K^{c}_{2}$ from $H[b_{2}]$ requires determining which is the symbol of $W=K^{c}_{1}$ that does not belong to $K^{c}_{2}$, removing it, and adding the missing DNA symbol from $H[b_{j+1}]$ to *W*. The reconstruction of $K^{c}_{1}$ from $H[b_{1}]$ might require visiting more than *k* buckets as we call *canspell* (the reason is in “Implementing our *k*-mer retrieval algorithm” section). However, after we get $K^{c}_{1}$, we obtain the subsequent *k*-mers from $b_{k'}, \ldots , b_{2}$ by visiting only one new bucket for each.

## Experiments

### Implementation details

We implemented^2^GetCanDict (“Building the canonical dictionary” section) and the variant of DumpDict that processes the canonical compact hash table (“Canonical scheme” section) in C++. We refer to this software as Kaarme. We did not implement GetDict and the variant of DumpDict that deals with the non-canonical hash table because most genomic analyses only use the canonical dictionary. Our source code implements the function *incval* in GetCanDict using compare and swap (CAS) atomic instructions to record the *k*-mers of $\mathcal {R}$ in parallel in a lock-free manner. To make the procedure more space efficient, we included a filtering step so GetCanDict can ignore most of the *k*-mers that do not appear at least twice. More specifically, we use two Bloom filters [3] where the first Bloom filter includes all *k*-mers occurring at least once in the data set, and the second Bloom filter includes all *k*-mers occurring at least twice in the data set. Thus, when first encountering a *k*-mer, we add it to the first Bloom filter. If a *k*-mer is already found in the first Bloom filter, we add it to the second Bloom filter. Only *k*-mers that are found in the second Bloom filter are added to the hash table of Kaarme. Because Bloom filters allow false positives, some *k*-mers with a single occurrence can be inserted into the hash table, but these are easily filtered out in the end when reporting the *k*-mers.

### Competitor tools

We compared our software (Kaarme) against the following methods:

1. Plain: a multi-threaded *k*-mer counter that uses a generic lock-free hash table implemented by us. The hash table stores the full *k*-mer sequences in a two-bits-per-symbol format, along with the frequencies.
2. Jellyfish [18]: a *k*-mer counter using a multi-threaded lock-free hash table.
3. CHTKC [31]: a semi-external *k*-mer counter. When the hash table is full, CHTKC stores all subsequent new *k*-mers on disk to be processed in a later batch.
4. DSK [26]: a disk-based *k*-mer counter that partitions the input and stores the partitions on disk.
5. Gerbil [9]: a *k*-mer counter with GPU support designed to efficiently count *k*-mers for large *k*.

We implemented Plain as a module within Kaarme. We use the flag -m to tell our software to either use our compact hash table scheme or Plain. Additionally, Plain and Kaarme require the user to estimate the number of distinct *k*-mers in the data set for them to calculate the bloom filter size. We computed the estimate by running DSK on the datasets to obtain the number of distinct *k*-mers. Jellyfish also requires an estimate of the number of distinct *k*-mers, so we gave it the value reported by DSK. CHTKC requires the user to define the maximum amount of memory it is allowed to use. We set this value to 15 GB, close to the maximum available memory of the used machine.

### Datasets

We used three read collections for the experiments:

1. ecoli280: 280x coverage PacBio HiFi *Escherichia coli* reads (acc: SRR10971019).
2. ecoli100: 100x coverage downsampled version of ecoli280.
3. dmel20: a downsampled 20x coverage PacBio HiFi *Drosophila melanogaster* reads (acc: SRR10238607).

We obtained the reads from the SRA^3^ database. See the associated accession codes in the list above.

### Experimental setup

**Fig. 5:**
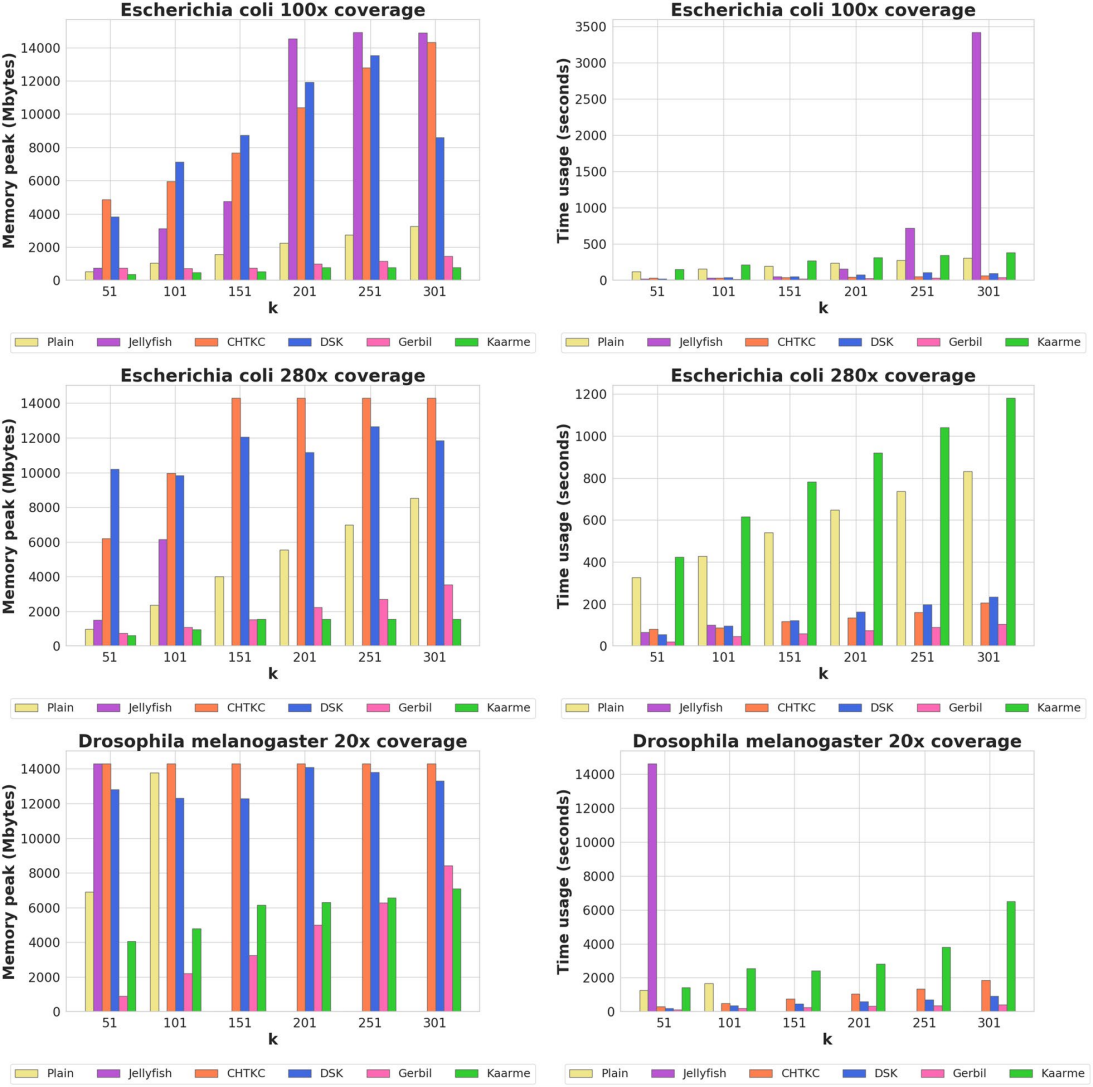
Memory usage (left column) and runtime (right column) of the tools on the ecoli100 (top row), ecoli280 (middle row) and dmel20 (bottom row) data sets. Missing columns indicate that *k*-mers could not be counted using the program

We used Kaarme and the competitors to count *k*-mers in the three data sets. The values of *k* we used were 51, 101, 151, 201, 251, and 301. The tools used up to 8 threads and reported canonical *k*-mers with frequency $\ge 2$. Memory usage was measured using time -v. This was also used to measure *k*-mer counting times of DSK, CHTKC, Jellyfish, and Gerbil. For Plain and Kaarme an internal timer was used. The run time and memory usage is shown in Fig. 5. Missing results indicate that a tool could not count the *k*-mers with the available amount of memory.

**Fig. 6:**
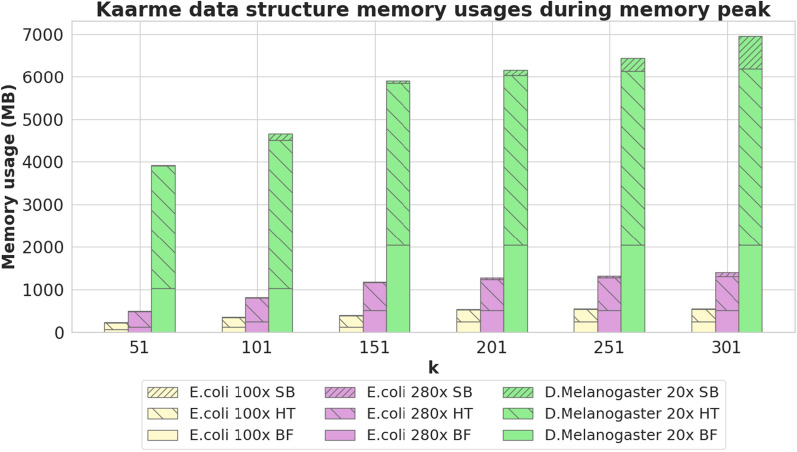
Estimated memory usage of the three main data structures of Kaarme during memory peak. SB = secondary buffer, HT = hash table, BF = bloom filter. Bloom filter size is doubled during filtering but is then halved when the unneeded first filter is deleted

**Fig. 7:**
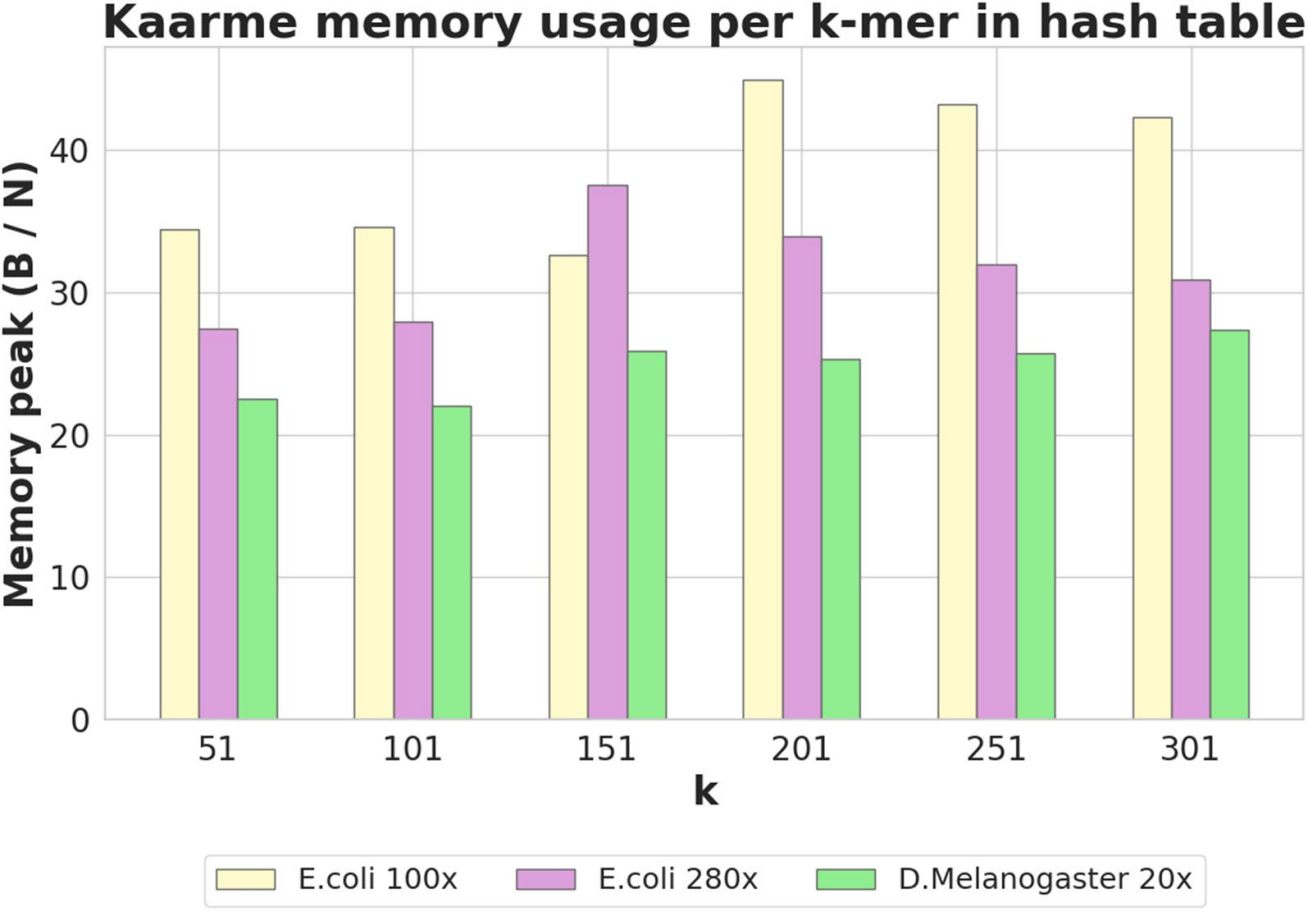
Memory usage of Kaarme per distinct *k*-mer stored in the hash table. (The number of *k*-mers in the hash table is close to the number of reported *k*-mers. Few with count = 1 slip through the bloom filter.)

**Fig. 8:**
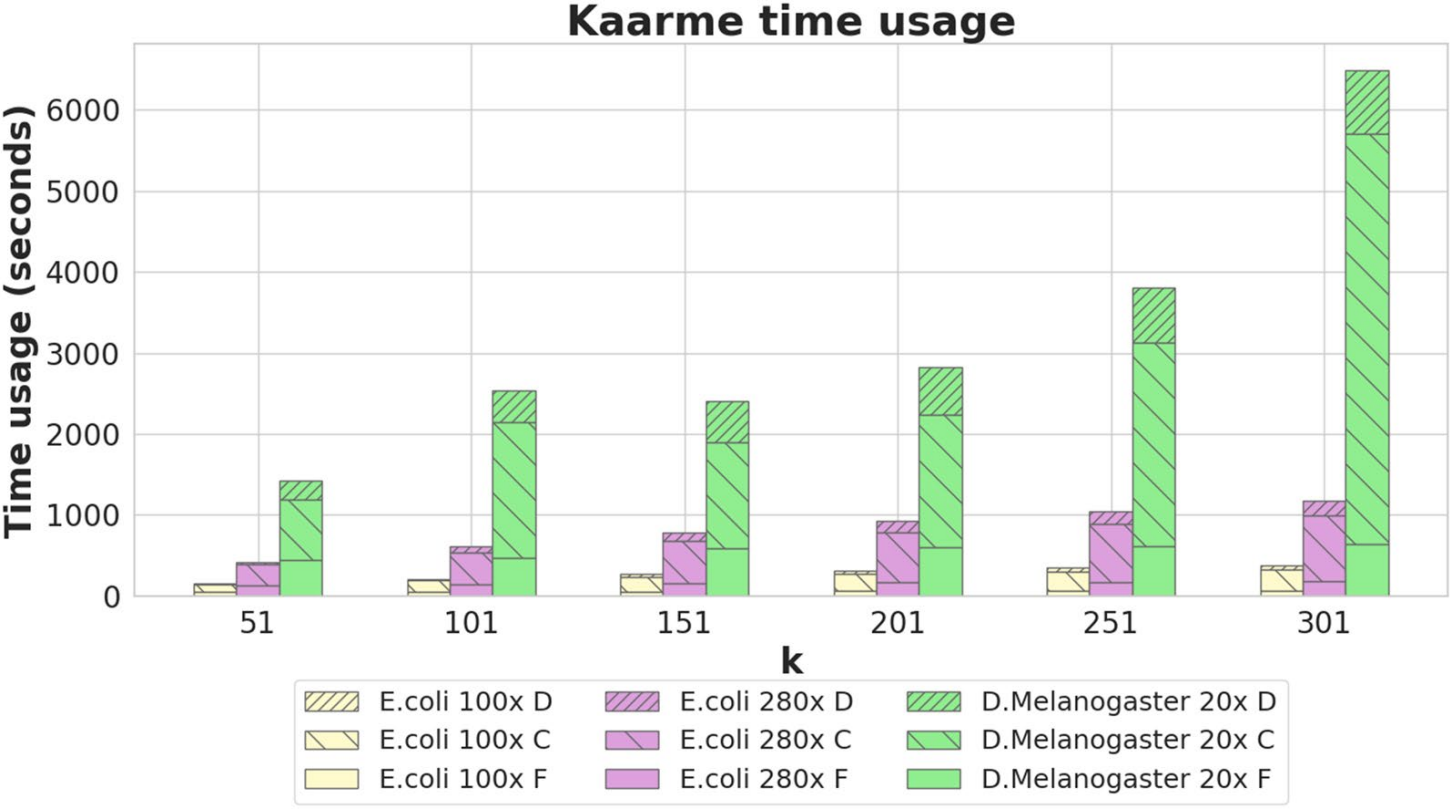
Kaarme has three phases: bloom filtering (F), counting (C), and dumping (D). This plot shows how much time each phase takes with the three different data sets

To illustrate the difference in memory usage for different values of *k*, statistics of Kaarme memory usage per distinct *k*-mer in the hash table can be seen in Fig. 7. To show how much time each procedure of Kaarme takes, in Fig. 8, the run times are split into three different parts: Bloom filtering, counting, and dumping. Figure 6 shows the estimated memory usages of the main Kaarme data structures. Note that the Bloom filter size includes both Bloom filters, which is halved by deleting the first bloom filter before we proceed to the *k*-mer counting step.

**Fig. 9:**
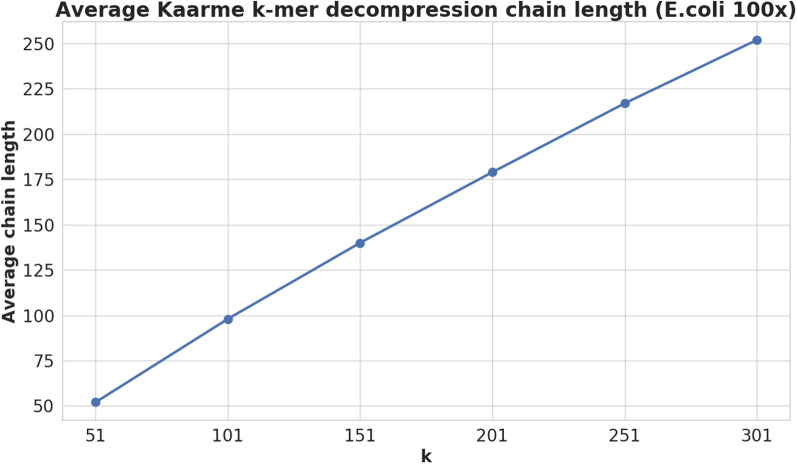
The average number of buckets visited when decompressing a *k*-mer in the canonical dictionary built on the ecoli100 data set

Finally, we measured the average number of buckets visited when decompressing a *k*-mer in the canonical dictionary built on the ecoli100 data set. We first constructed the canonical dictionary $\mathcal {D}_{k,\mathcal {R}}^{c}$. Then, *k*-mers on the occupied entries of the hash table were decompressed, and the number of visited buckets during decompression was recorded. The average lengths of these *k*-mer decompression chain lengths are shown in Fig. 9.

The experiments were run on a laptop with 16 GB of RAM, Intel$\circledR$ Core^TM^ i5-8250U CPU @ 1.60GHz $\times$ 8 processor, and a 64-bit Linux-based OS.

## Results and discussion

First, we compare Kaarme to Plain. Figure 5 shows that Kaarme uses significantly less memory than Plain. On ecoli100 with $k=51$, the space usage of Kaarme is about 70% of the space usage of Plain (362MB vs 529MB), and the difference grows as the value of *k* increases. On ecoli280, the difference is even more significant with larger values of *k*. On dmel20 with $k=51$, Kaarme also uses about 40% less memory than Plain, and Kaarme could run with *k* up to 301 while Plain ran out of RAM already when *k* was set to 151. However, the reduced space usage does not come completely without a cost. The runtime of Kaarme is longer compared to Plain (for example, 151 s vs 122 s with $k=51$ on ecoli100).

In all the datasets, the space usage of Kaarme was dominated by the hash table and Bloom filters, while the secondary buffer took on average less than 11% of the total space usage (see Fig. 6). We remark that our hash table’s compact encoding uses a constant number of bits per *k*-mer (regardless of *k*), and that our experiments showed that the secondary buffer contributes little to the memory peak. Therefore, we expect the space usage per distinct *k*-mer to grow very slowly with Kaarme, and to grow linearly with *k* for Plain. This is indeed the case as shown in Fig. 7. On the other hand, Fig. 8 shows that the running time of Kaarme is dominated by the counting phase, especially when *k* grows.

We see in Fig. 5 that, when compared to other *k*-mer counters, Kaarme uses the least amount of memory in all other experiments except on dmel20, where Gerbil is more memory efficient with $k<300$. However, Kaarme is slower than the other *k*-mer counters with the exception of Jellyfish on some data sets where its memory usage is close to the total RAM available on the machine.

We remark that Kaarme only implements compact in-memory hash tables that are suitable for *k*-mers, while our competitors are full-fledged counters that combine hash tables with other techniques. Thus, a comparison of Kaarme against these tools is not completely fair. This observation is particularly true for Gerbil, CHTKC, and DSK that rely on disk to reduce RAM usage.

Gerbil, DSK, and CHTKC control the amount of main memory they use, so they can count the *k*-mers in all the data sets without running out of RAM. CHTKC and DSK used RAM up to the set limit of 15GB but made exhaustive use of disk when it was deemed necessary. Because of the disk usage, the comparison between all the programs is not strictly fair. Still, the space usage of Kaarme was usually the smallest, excluding lower *k* experiments on dmel20, indicating that Kaarme is the most memory frugal.

## Concluding remarks

We have presented Kaarme, a space-efficient hash table to count large *k*-mers in memory. We showed that Kaarme uses up to five times less space than a regular hash table for counting *k*-mers while being at most 1.5 times slower. When compared to *k*-mer counters, Kaarme uses the least amount of memory when *k* is large.

We note that both DSK and CHTKC make use of hash tables in their implementation. Thus, the adaption of Kaarme as a submodule in these tools could allow them to either use less memory or count larger *k*-mer sets in memory. However, Kaarme takes advantage of the fact that most of the *k*-mers overlap by $k-1$ symbols with a previous *k*-mer in the input collection. Depending on how DSK partitions the *k*-mers, the input data set could become more fragmented with a much larger amount of *k*-mers without predecessors, causing the secondary buffer *B* to grow significantly. The same could be true for CHTKC, which writes the excess *k*-mers into disk for the next iteration.

The reconstruction of *k*-mers in the canonical dictionary of Kaarme can be exponential, but our experiments suggest that, on average, the time complexity seems to be close to linear. Therefore, Kaarme is a practical, space-efficient hash table for large *k*-mers.

## Data Availability

The datasets for the experiments were obtained from public repositories, while the source code is publicly available on GitHub. See the corresponding links in “Our contribution” section (Experiments).

## Footnotes

1 Käärme is a snake in Finnish. The way the *k*-mers are arranged in the hash table resembles a snake weaving its way through the table.

2 https://github.com/Denopia/kaarme.

3 https://www.ncbi.nlm.nih.gov/sra.
